# Uterus-preserving surgical management of placenta accreta spectrum disorder: a large retrospective study

**DOI:** 10.1186/s12884-023-05923-9

**Published:** 2023-08-26

**Authors:** Wenxia Pan, Juan Chen, Yinrui Zou, Kun Yang, Qingfeng Liu, Meiying Sun, Dan Li, Ping Zhang, Shixia Yue, Yuqiang Huang, Zhaoxi Wang

**Affiliations:** 1 Department of Obstetrics, Linyi Maternal and Child Healthcare Hospital, NO.1, South Qinghe Road, Luozhuang District, Linyi City, 276016 Shandong Province China; 2 Havy International (Shanghai) Ltd, Building 25, No.1665, Kongjiang Road, Yangpu District, Shanghai, 200092 China; 3 Department of Radiology, Linyi Maternal and Child Healthcare Hospital, NO.1, South Qinghe Road, Luozhuang District, Linyi City, 276016 Shandong Province China; 4 Department of Ultrasound, Linyi Maternal and Child Healthcare Hospital, NO.1, South Qinghe Road, Luozhuang District, Linyi City, 276016 Shandong Province China; 5 Department of Nursery, Linyi Maternal and Child Healthcare Hospital, NO.1, South Qinghe Road, Luozhuang District, Linyi City, 276016 Shandong Province China; 6 Department of Pediatric Cardiology, Linyi Maternal and Child Healthcare Hospital, NO.1, South Qinghe Road, Luozhuang District, Linyi City, 276016 Shandong Province China; 7 Beth Israel Deaconess Medical Center, Harvard Medical School, 330 Brookline Avenue, Kirstein 3, 02215 Boston, MA USA

**Keywords:** Placenta accreta spectrum, Placenta previa, Conservative management, Uterus preservation

## Abstract

**Background:**

The two-child policy implemented in China resulted in a surge of high-risk pregnancies among advanced maternal aged women and presented a window of opportunity to identify a large number of placenta accreta spectrum (PAS) cases, which often invoke severe blood loss and hysterectomy. We thus had an opportunity to evaluate the surgical outcomes of a unique conservative PAS management strategy for uterus preservation, and the impacts of magnetic resonance imaging (MRI) in PAS surgical planning.

**Methods:**

Cross-sectional study, comparing the outcomes of a new uterine artery ligation combined with clover suturing technique (UAL + CST) with the existing conservative surgical approaches in a maternal public hospital with an annual birth of more than 20,000 neonates among all placenta previa cases suspecting of PAS between January 1, 2015 and December 31, 2018.

**Results:**

From a total of 89,397 live births, we identified 210 PAS cases from 400 singleton pregnancies with placenta previa. Aside from 2 self-requested natural births (low-lying placenta), all PAS cases had safe cesarean deliveries without any total hysterectomy. Compared with the existing approaches, the evaluated UAL + CST had a significant reduction in intraoperative blood loss (β=-312 ml, *P* < .001), RBC transfusion (β=-1.08 unit, *P* = .001), but required more surgery time (β = 16.43 min, *P* = .01). MRI-measured placenta thickness, when above 50 mm, can increase blood loss (β = 315 ml, *P* = .01), RBC transfusion (β = 1.28 unit, *P* = .01), surgery time (β = 48.84 min, *P* < .001) and hospital stay (β = 2.58 day, *P* < .001). A majority of percreta patients resumed normal menstrual cycle within 12 months with normal menstrual fluid volume, without abnormal urination or defecation.

**Conclusions:**

A conservative surgical management approach of UAL + CST for PAS is safe and effective with a low complication rate. MRI might be useful for planning PAS surgery.

**Clinical trial registration number:**

: ChiCTR2000035202.

**Supplementary Information:**

The online version contains supplementary material available at 10.1186/s12884-023-05923-9.

## Background

Placenta accreta spectrum (PAS) is pathologic adherence and excessive penetration of part or all of the placenta into the myometrium, including accreta, increta, or percreta [1–3]. Placenta previa, prior cesarean delivery (CD), uterine surgery, multiparity, advanced maternal age, as well as in vitro fertilization are risk factors associated with the worldwide increase in PAS [4–7]. PAS can induce massive hemorrhage as the placenta cannot separate spontaneously at delivery, which often requires cesarean hysterectomy to control serious bleeding. The intraoperative blood loss is reported to range from 2,000 to 5,000 ml, and frequently blood transfusion is needed. In severe cases, PAS can cause maternal death, with mortality rate as high as 6–7% [8, 9].

The management of PAS include early prenatal screening and referral to tertiary centers with experienced multidisciplinary teams [10–12]. Obstetrical ultrasound in the second or third trimester is the primary method for the screening and diagnosis of PAS, but it suffers from high inter-operator variability and low reproducibility, and it cannot obtain a panorama view of placenta [13]. With better visualization of pelvic organs and additional details of the utero-placental relationship, magnetic resonance imaging (MRI) has also been adopted as an antenatal diagnostic tool [14]. Currently, there are still controversies regarding the benefits of MRI, particularly given the increased cost of MRI.

According to International Federation of Gynecology and Obstetrics (FIGO) guidelines, the principal surgical strategy to prevent excessive bleeding related to PAS is to leave the placenta in situ and perform a primary peripartum hysterectomy at delivery [15]. A hysterectomy may be not preferred by patients wishing to preserve fertility and is detrimental to multiple aspects of pelvic floor, bowel and physical functions [16–18]. Moreover, in some cultures, the removal of her uterus may reduce a woman’s societal status and therefore negatively impact her self-esteem [15].

After China raised a family’s limit on children to two at the end of 2015, there was an increase in second pregnancies, and the incidence of PAS and associated maternal deaths dramatically increased across the country [19]. As the only tertiary referral center for maternal and child healthcare in Linyi City, Shandong, China, a major metropolitan area with a population of 11 million people, the study center has accumulated a large number of PAS cases. This region believes in the traditional Chinese value that multiple children are fundamental to family happiness and harmony. Therefore, conservative approach of preserving uterus is commonly implemented in obstetric practice here. Gradually, an obstetric surgeon developed a technique, which combines uterine artery ligation with clover suture technique (UAL + CST) together. This technique prevents excessive bleeding and preserves uterus. Meantime, other surgery teams continued with existing compression sutures including B-lynch suture, modified B-lynch suture, CHO suture and row suture [20–23].

The purpose of this study was to compare the UAL + CST approach and other existing approaches by measuring blood loss using direct measurement and gravimetric methods, blood transfusion, and other adverse effects to determine the feasibility of UAL + CST approach. The secondary purpose was to evaluate the impact of MRI in PAS management, as it was commonly implemented in obstetric practice in this center.

## Methods

This retrospective study was conducted at Linyi Maternal and Child Healthcare Hospital, Shandong, China, one of the largest hospitals by annual live births in China. This study was approved by the Institutional Review Board of Linyi Maternal and Child Healthcare Hospital. Subjects were eligible for inclusion if they delivered a live birth between January 1, 2015 and December 31, 2018 in the study center.

In chart review, a physician team manually screened electronic medical records for a diagnosis of placenta previa. Then, PAS cases were identified by reviewing ultrasound reports, MRI reports, and surgical reports from previa cases. Twin pregnancies were excluded. PAS cases were classified into four groups including accreta (grade 1), increta (grade 2), and percreta (grade 3), according to FIGO clinical classification [1].

This center has a routine ultrasonographic screening program for placenta previa. An ultrasonography is performed monthly in accordance with the recommendations from FIGO [24]. If any ultrasound exam suggests placenta previa, the patient is transferred to the dedicated outpatient service. If PAS is suspected at approximately 30 weeks of gestation, obstetricians would prescribe an MRI examination using a modified protocol [25].

**Fig. 1:**
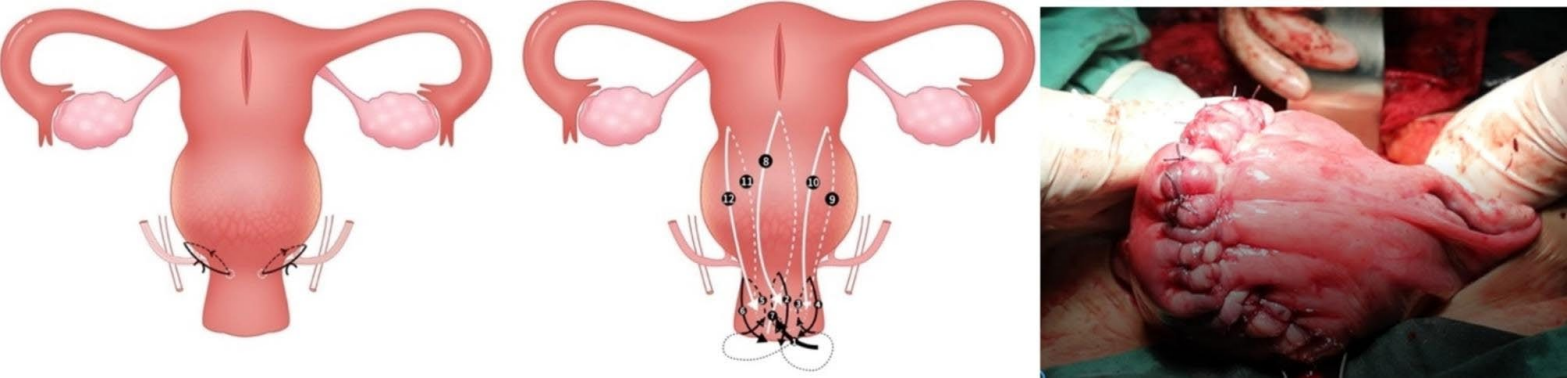
**a**. Schematic diagram of uterine artery ligation (UAL); **b**. Schematic diagram of clover suturing technique (CST); **c**. Picture of a uterus after UAL + CST

The elections of the UAL + CST surgery or other existing conventional approaches, including B-lynch suture, modified B-lynch suture, CHO suture, row suture and hysteroplasty, was not based on preoperative assessment but on the schedule of surgeons and could not be switched intraoperatively. In the UAL + CST approach, four surgical steps were performed thereafter. First, the bladder was pushed down to ligate invading blood vessels as the plane between the bladder and the uterus is relatively clear at that stage, and therefore easier to identify. Second, lift the upper edge of the abdominal incision with an abdominal wall hook, and a vertical or horizontal incision was made on the upper uterus. After delivering the fetus, while avoiding touching the placenta, the umbilical cord was cut and oxytocin is injected into the body of uterus. Third, at 1 centimeter inside the interface of uterine artery and lateral margin of uterus, the ascending branch of uterine artery was ligated together with some myometrium tissue, the ligation thread and the lateral margin of uterus forming a 30 degree angle, avoiding the ureter (Fig. 1a). Fourth, the placenta was extracted manually, and CST (Fig. 1b) was performed on the lower uterus. CST was proposed by the team specifically for PAS surgery. It was usually performed on the lower segment of the uterus, and also on the cervix when the placenta invasion reached the cervix, during which cervical CST proceeds uterine CST. Figure 1b shows the condition when CST was performed both on the cervix and the lower segment of the uterus, and Fig. 1c is a picture when CST was done. (For detailed description of CST, see supplement material 2). Partial hysterectomy (excision of partial uterine wall) was performed when it was deemed too difficult to separate the placenta manually. For detailed surgery process please refer to supplement material 1.

MRI was widely used for close examination of patients’ utero-placenta structure and surgical planning in this center. For objective evaluation of the impact of MRI, we measured the placenta thickness and used this parameter in data analysis.

We also prospectively followed the most severe 19 cases of percreta (grade 3), including their gynecological examinations, emergency care, menses recovery, and incidences of abdominal pain, abnormal urination and defecation. Written informed consents were obtained.

Epidata was used for data collection and SPSS 25.0 package was used for data analysis. Continuous values were presented as mean ± SD, or median (interquartile range). Categorical values were expressed as counts (percentage). For continuous variables, Kolmogorov–Smirnov analysis was applied to test distribution. If normally distributed, independent *t* test was performed; if skewed distributed, logarithmic conversions were performed and the distribution was tested again; if still skewed distributed, the Mann Whitney U test was performed. For categorical variables, we performed the Chi Square test or Fisher exact test. Multivariate linear regression analysis was also performed. *P* < .05 at both sides was considered to be statistically significant.

## Results

During the four-year study period, there was a total of 89,397 live births at the study center, of which 400 singleton placenta previa cases were identified (Table 1). Specifically, 210 cases complicated with PAS, including 107 accreta cases (grade 1), 84 increta (grade 2) cases, and 19 percreta cases (including 11 grade 3a and 8 grade 3b).

**Table 1:** Demographic characteristics

	Accreta			Increta			Percreta		
Variable	UAL + CST	Existing Approach	*P*	UAL + CST	Existing Approach	*P*	UAL + CST	Existing Approach	*P*
	(N = 44)	(N = 63)		(N = 60)	(N = 24)		(N = 15)	(N = 4)	
Age, yrs ^a^	32.0 (28.0-38.8)	31.0 (27.0–36.0)	0.23	32.0 (30.0–36.0)	32.0 (28.0-35.5)	0.71	30.0 (29.0–34.0)	33.0 (28.8–35.0)	0.64
Gestation age at delivery, wks ^a^	37.4 (36.6–38.3)	37.1 (35.0-38.3)	0.47	36.9 (35.7–37.6)	37.1 (36.1–38.1)	0.33	36.7 (35.1–37.6)	37.5 (28.0-38.9)	0.47
Gravidity, n (%) ^b^			0.32			0.78			1
≤ 4	33 (75)	53 (84)		46 (77)	18 (71)		10 (67)	3 (75)	
> 4	11 (25)	10 (16)		14 (23)	7 (29)		5 (33)	1 (25)	
Parity, n (%) ^b^			0.23			0.28			0.3
≤ 1	41 (93)	53 (84)		46 (77)	15 (63)		6 (40)	3 (75)	
> 1	3 (7)	10 (16)		14 (23)	9 (37)		9 (60)	1 (25)	
Number of previous CD, n (%) ^c^			**0.049**			0.52			0.68
0	26 (59)	50 (79)		20 (33)	6 (25)		1 (6))	0	
1	16 (36)	12 (19)		31 (52)	12 (50)		7 (47)	3 (75)	
2	2 (5)	1 (2)		9 (15)	6 (25)		7 (47)	1 (25)	
APGAR scores < 7, n (%)									
At 1 min ^c^	2 (5)	5 (8)	0.7	4 (7)	2 (8)	1	1 (100)	0	1
At 5 min ^c^	0	0	N/A	1 (2)	0	1	0	0	NA
Delivery weight, g^a^	3080 (2850–3443)	3028 (2663–3390)	0.3	2970 (2600–3330)	3050 (2650–3430)	0.33	2751 ± 478	3463 ± 270	**0.03** ^d^
Placenta thickness, mm ^a^	36.7 (33.1–41.8)	38.0 (31.0–43.0)	0.91	39.3 (33.0-50.8)	37.5 (32.8–44.8)	0.51	43.0 (31.6–48.8)	36.5 (32.3–56.8)	0.871
MRI, n (%) ^b^	22 (50)	11 (18)	**0.001**	35 (58)	11 (46)	0.34	11 (73)	3 (75)	1
Surgery type, n (%) ^c^			0.65			0.06			1
Elective surgery	23 (52)	27 (43)		31 (52)	12 (50)		10 (67)	3 (75)	
Gestation age at delivery, wks ^a^	37.4 (37.1–38.6)	37.7 (36.7–39.0)	0.653	37.1 (36.4–37.6)	37.3 (36.5–38.5)	0.457	36.7 ± 1.5	38.0 ± 1.1	0.169 ^d^
Inpatient emergency surgery	9 (21)	16 (25)		6 (10)	7 (29)		0	0	
Gestation age at delivery, wks ^a^	36.9 (36.5–37.9)	36.2 (33.4–38.1)	0.607	35.7 ± 2.3	36.3 ± 1.8	0.598^d^	NA	NA	NA
Outpatient emergency surgery	12 (27)	20 (32)		23 (38)	5 (21)		5 (33)	1 (25)	
Gestation age at delivery, wks ^a^	36.7 (35.2–38.3)	36.1 (30.6–38.0)	0.402	36.1 (32.6–37.1)	36.9 (35.0–38.0)	0.455	34.9 ± 3.1	25.0	NA

Values are given as median (interquartile range), mean ± SD or number of subjects (percentage), unless indicated otherwise

Gravidity, parity and number of previous CD values are before the studied delivery values

CD: cesarean delivery; APGAR: activity, pulse, grimace, appearance and respiration; MRI: magnetic resonance imaging; SD: standard deviation

^a^ Mann Whitney U test

^b^ Chi-square test

^c^ Fisher’s exact test

^d^ Independent T test

Excluding 2 cases delivered vaginally (low-lying placenta, self-requested vaginal delivery), 208 PAS cases had CD with 106 elective surgery (50.5%) and 104 emergency surgery (49.5%) (Table 1). For accreta group, patients received UAL + CST generally had more previous CD than those having the existing approaches (*P* = .05). For percreta group, newborn birth weight in UAL + CST surgery group were significantly lower than those using the existing approaches (*P* = .03). Additionally, the surgeon using UAL + CST in accreta groups were more likely to employ MRI to plan surgery (*P* = .001, respectively). Other demographic characteristics of all groups were comparable.

There was no total cesarean hysterectomy performed on any subject, but 33 (15%) PAS cases had partial hysterectomies (excision of partial uterine wall) mostly associated with deep PAS penetration, including 25% of increta cases and 63% of percreta cases (Table 2). Though data did not show significant difference, the UAL + CST approach needed less partial hysterectomy (excision of partial uterine wall) than the existing approaches. Additionally, there were only 6 (1.5%) puerperal infections among all groups, and 4 (21%) bladder injuries in the percreta group. None of the adverse effects showed significant difference between the two surgical approaches in all groups. However, UAL + CST showed significant protective effect in terms of lower operative blood loss (*P* = .01) and need for transfusion *(P* = .05) in increta group than conventional approaches. Besides, the UAL + CST did require more surgery time as suggested in accreta (*P* = .001) and increta (*P* = .02) groups. In contrast, the UAL + CST only had an average of ~ 15 min increase of surgical time (*P* = .001) in the accreta cases. Considering the small number of percreta cases, the UAL + CST had a lower average intraoperative blood loss but only at a marginal significance.

**Table 2:** Surgery Outcomes

	Accreta			Increta			Percreta		
Outcome	UAL + CST	Existing Approach	*P*	UAL + CST	Existing Approach	*P*	UAL + CST	Existing Approach	*P*
	(N = 44)	(N = 63)		(N = 60)	(N = 24)		(N = 15)	(N = 4)	
Intraoperative blood loss (mL) ^a^	300 (300–500)	400 (300–500)	0.17	500 (300–800)	800 (500–2000)	**0.01**	600 (500–1000)	2000 (850–3375)	0.052
Intraoperative blood loss > 1,500mL^c^	1 (1)	0	0.41	3 (5)	7 (29)	**0.01**	3 (20)	2 (50)	0.27
Intraoperative RBC transfusion (u) ^a^	0.00 (0.00–0.00)	0.00 (0.00–0.00)	0.37	0.00 (0.00-3.88)	2.00 (0.00–4.00)	**0.048**	2.00 (0.00–4.00)	4.00 (4.00-12.63)	0.12
Massive RBC transfusion (≥ 6u) ^c^	0	0	NA	1 (2)	4 (17)	**0.02**	3 (20)	1 (25)	1
Any RBC transfusion ^b^	4 (9)	10 (16)	0.39	21 (35)	12 (50)	0.225	9 (60)	4 (100)	0.26
Surgery duration (min) ^a^	64.0 (50.0-79.5)	50.0 (45.0–60.0)	**0.001**	110.0 (75.5–145.0)	75.5 (53.5-122.5)	**0.02**	187.3 ± 79.2	180.7 ± 69.7	0.88^d^
Hysterectomy	0	0	NA	0	0	NA	0	0	NA
Postoperative hospital stays (day) ^a^	4.0 (3.0–5.0)	4.0 (3.0–4.0)	0.48	5.0 (4.0–7.0)	4.5 (4.0–6.0)	0.15	7.0 (6.0–15.0)	11.0 (6.3–15.8)	0.59
Puerperal infection ^c^	0	1 (2)	1	2 (3)	0	1	0	1 (25)	0.21
Bladder Injury	0	0	NA	0	0	NA	3 (20)	1 (25)	1
Ureteral Injury	0	0	NA	0	0	NA	0	0	NA
Intestinal Injury	0	0	NA	0	0	NA	0	0	NA
Placenta remains	0	0	NA	0	0	NA	0	0	NA
Reoperation ^c^	0	0	NA	1 (2)	0	1	0	0	NA
Partial hysterectomy ^c^	0	0	NA	14 (23)	7 (29)	0.78	8 (53)	4 (100)	0.25
Partial bladder resection ^c^	0	0	NA	0	0	NA	2 (13)	1 (25)	0.53

Values are given as median (interquartile range), mean ± SD or number of subjects (percentage), unless indicated otherwise

^a^ Mann Whitney U test

^b^ Chi-square test

^c^ Fisher’s exact test

^d^ Independent T test

We further conducted multivariate linear regression analyses with adjustments for age, number of previous CDs, gestational weeks, surgical approach, placental thickness (≥ 50 mm under ultrasound or MRI), and level of PAS penetration (Table 3). We found comparable increases in the intraoperative blood loss, RBC transfusion, surgery duration, and postoperative hospital stay for each level increase of PAS severity from accreta to percreta. When placenta thickness measured more than 50 mm in MRI was added into the model, it was associated with adverse outcomes i.e., more blood loss, RBC transfusion, longer surgery time and hospital stay. The UAL + CST surgical approach significantly reduced the intraoperative blood loss (β=-312 ml, *P* < .001) and RBC transfusion (β=-1.08 unit, *P* = .001). Although this technique necessitated more surgery time (β = 16.43 min, *P* = .01), it conferred no significant changes in postoperative hospital stay.

**Table 3:** Multivariate linear regression models (N = 210)

	Model 1 (excluding placenta thickness)			Model 2 (including placenta thickness)		
	β	95%CI	*P* Value	β	95%CI	*P* Value
**Intraoperative Blood Loss (ml)**						
Age	3	-8–14	0.63	6	-6–18	0.33
Previous C-section	143	35–251	**0.01**	105	-17–226	0.09
Gestation Week	3	-7–14	0.54	0	-12–12	1
UAL + CST ^a^	-312	-449 - -176	**< 0.001**	-387	-538 - -237	**< 0.001**
PAS	313	198–429	**< 0.001**	332	206–458	**< 0.001**
Placenta thickness	-	-	-	315	99–530	**0.01**
**Intraoperative RBC Transfusion (unit)**						
Age	0.01	-0.04–0.06	0.76	0.03	-0.03–0.08	0.36
Previous C-section	0.85	0.37–1.34	**0.001**	0.51	-0.02–1.03	0.06
Gestation Week	-0.03	-0.08–0.02	0.25	-0.05	-0.10–0.00	0.07
UAL + CST ^a^	-1.08	-1.69 - -0.46	**0.001**	-1.08	-1.73 - -0.44	**0.001**
PAS	1.32	0.81–1.84	**< 0.001**	1.35	0.80–1.89	**< 0.001**
Placenta thickness	-	-	-	1.28	0.35–2.21	**0.01**
**Surgery Duration (min)**						
Age	0.67	-0.29–1.62	0.17	1.2	0.17–2.24	**0.02**
Previous C-section	31.43	22.09–40.77	**< 0.001**	31.2	20.92–41.48	**< 0.001**
Gestation Week	-0.5	-1.41–0.41	0.28	-0.95	-1.93–0.04	0.06
UAL + CST ^a^	16.43	4.68–28.19	**0.01**	13.76	1.07–26.45	**0.03**
PAS	37.57	27.62–47.53	**< 0.001**	33.89	23.26–44.53	**< 0.001**
Placenta thickness	-	-	-	48.84	30.64–67.04	**< 0.001**
**Postoperative Hospital Stay**						
Age	0.05	-0.02–0.12	0.15	0.09	0.02–0.17	**0.02**
Previous C-section	0.92	0.24–1.61	**0.01**	0.99	0.22–1.76	**0.01**
Gestation Week	0	-0.07–0.07	1	-0.03	-0.10–0.05	0.48
UAL + CST ^a^	0.27	-0.59–1.14	0.53	-0.11	-1.06–0.84	0.82
PAS	1.81	1.08–2.53	**< 0.001**	1.52	0.72–2.31	**< 0.001**
Placenta thickness	-	-	-	2.58	1.22–3.94	**< 0.001**

^a^ UAL + CST: uterine artery ligation combined with CST.

Placenta thickness < 50 mm was deemed as 0 and placenta thickness ≥ 50 mm was deemed as 1 in model 2

Next, we conducted a follow-up evaluation of all percreta cases, which is the most severe form of PAS and often has serious and complicated sequelae (Table 4). Out of 19 patients, 5 (26%) patients couldn’t be reached, the average duration of follow-up for the 14 respondents was 20.3 months after delivery, ranging from 10.9 months to 37.8 months. Twelve respondents completed a follow-up gynecological examination, and one case was noted to have abnormal intrauterine adhesions. The majority of these percreta patients (n = 10) resumed a normal menstrual cycle within 12 months from delivery, with an average of 4.8 months (range 1–12 months). There were 3 (21%) patients didn’t resume menstrual cycle, with 1 patient still under breast feeding (censored time: 11 month). Most patients reported normal menstrual fluid volume, with only one case having reduced menstrual fluid volume compared to prior delivery. Additionally, there was one case with dysmenorrhea that existed prior to pregnancy. None of the 14 cases reported abnormal urination or defecation.

**Table 4:** Follow-up results of severe percreta cases

Subject	Censored Time (month)	Gynecological examination	Gynecological disease	Mense Resumption ^a^	Mense resumption time ^b^	Menses Volume	Abdominal pain	Abnormal urination/ defecation
1	37.8	Yes	No	Yes	2	Normal	No	No
2	37.6	No	/	Yes	1	Normal	No	No
3	36.1	Yes	No	Yes	4	Normal	No	No
4	35.4	Yes	Yes ^c^	No	/	/	No	No
5	33.4	Yes	No	Yes	3	Normal	No	No
6	30.8	Yes	No	No ^d^	/	/	No	No
7	17.5	Yes	No	Yes	3	Normal	No	No
8	16.1	No	/	Yes	6	Normal	No	No
9	13.8	Yes	No	Yes	1	Normal	No	No
10	13.0	Yes	No	No	/	/	No	No
11	10.9	Yes	No	No	/	/	No	No
12	36.0	Yes	No	Yes	12	Decreased	No	No
13	34.2	Yes	No	Yes	8	Normal	Dysmenorrhea^e^	No
14	33.8	Yes	No	Yes	8	Normal	No	No

^a^ Resumption of normal menstrual cycle

^b^ Duration before resumption of normal menstrual cycle since delivery (month)

^c^ Intrauterine adhesions

^d^ Breast-feeding

^e^ Existed prior to delivery

We found that three studied patients, who were identified as PAS before, were pregnant again and delivered in the study center in 2021. Two of them had preterm C-section, the other one had full-term C-section.

## Discussion

Since PAS is a life-threatening condition often accompanied with postpartum hemorrhage or hysterectomy, cesarean hysterectomy with the placenta left in situ has been the conventional management approach [26]. However, cesarean hysterectomy is technically challenging, with a high maternal mortality due to massive hemorrhage, and surgical complications such as urinary tract, bowel, or pelvic nerve injuries, in addition to loss of fertility and its accompanying psychological trauma are not uncommon [9]. Moreover, this procedure is not suitable for patients and their families who are keen to preserve fertility.

In contrast to expectant management of leaving the placenta partially or totally in situ, several approaches of conservative management have been developed [27] as have adjunctive techniques for controlling hemorrhage, including pelvic devascularization, embolization, endouterine hemostatic suture, uterine compression suture, use of tissue sealants or mesh, uterine artery balloon placement, embolization or ligation, and postdelivery oxytocin administration [15, 28, 29]. Although randomized trials of conservative management in PAS cases are not available, several case series reported a reduced hysterectomy rate to ~ 20% of PAS patients [30, 31].

In this study, the prominent hallmark of surgical management was the preservation of the uterus without leaving the placenta in situ, which results in significantly less blood loss. In the past, some surgeons in our center performed prophylactic placement of internal iliac artery balloon catheters in conservative management of two PAS cases, but this unfortunately resulted in significant blood loss (data not shown), and therefore this technique was subsequently abandoned. Bilateral uterine artery ligation was later adopted for hemorrhage control combined with a dedicated suture technique. Therefore, the conservative management approach used by the surgical team was manual separation of the placenta combined with ligation of the ascending branch of uterine artery and clover suturing for hemorrhage control (UAL + CST), which has proved to be effective in preventing postpartum hemorrhage [32]. As a result, other surgery teams in the study center gradually adopted this approach.

When China allowed families to have two children instead of limiting to one, there was a 50% increase in annual live births in the study city, peaking in 2017 at 250,857 live births, the highest birth rate growth in China [33]. However, despite the national trend of increased maternal death and surge of PAS cases due to higher maternal age and history of prior CD, there were no maternal deaths related to PAS at this center, suggesting that an effective system of perinatal health management was established. Furthermore, despite a more conservative approach, compared to previous published outcomes of PAS surgical operations, this study suggests that this approach led to comparable or better patient outcomes than total hysterectomy [26, 30, 34, 35]. All women, excluding 3 vaginal deliveries, which were requested by patients themselves, had safe deliveries through elective or emergent cesarean sections. Even considering 18% of CDs were performed emergently, there was a reduction in the average blood loss (611 mL), which is far lower both than immediate hysterectomy of 3000 mL and delayed hysterectomy of 750 mL reported in previous research [36]. Besides, the transfusion rate ( 29%) was lower when compared with 34–78% as previously described [34]. The average operative time (92 min) and length of stay (5.3 days) were comparable to those reported in the literature [34]. Moreover, only a few postoperative complications were reported among 210 PAS cases, including 4 (2%) puerperal infections, 4 (2%) bladder injuries, and 1 (0.5%) follow-up operation. No ureteral injuries, intestinal injuries, or placental remnant were reported. Most importantly, there were no deaths and no total hysterectomies performed. Overall, the present study illustrates a low-risk surgical management strategy as reflected by the described outcomes.

This study demonstrated that the conservative approach of PAS management was safe and could preserve the uterus. In our follow-up with the most severe form of PAS, the majority of percreta patients (n = 10) resumed normal menstrual cycle within 12 months with normal menstrual fluid volume. As PAS is closely related to previous C-section and parity, most of the patients have had two or more than two children, and they usually would not consider fertility issue. The three patients who later delivered again could be the evidence that the studied surgery technique preserved not only the uterus but also the fertility.

Another distinctive finding of this study was the application of MRI in PAS management in a large proportion of cases (~ 44%). After excluding cases with emergency surgeries, which seldom had adequate time for MRI, the rate of MRI in PAS increased to 54%. It is well-known that MRI offers better visualization of maternal pelvic organs, particularly when abnormal invasion (increta and percreta) is suspected, and offers additional detail regarding the utero-placental relationship and the surrounding periuterine environment [14]. There are still controversies about the accuracy of MRI in PAS diagnosis when used as an adjunct to ultrasound [37, 38]. However, MRI in the study center was mainly used for surgical management of PAS, as MRI could provide greater spatial resolution of the entire placenta and presenting the relationships between the uterus and adjacent anatomic structures. Preoperative topology of PAS by MRI has been shown to enable better surgical planning, as it may predict the likelihood of bleeding, postoperative complications, and possibility of uterine repair [39].

It should be acknowledged that part of the reason for the high use of MRI in this study were due to the relatively low cost of MRI at the studied hospital, less than 100 US dollars. We did not find significant impact of use of MRI on blood loss, transfusions, operation durations, hospital stays, and complications. However, in our study, placenta thickness ≥ 50 mm was shown to be closely related to more blood loss and RBC transfusion and longer surgery time and hospital stay. As MRI could obtain a panorama view of placenta and find the thickest part, whereas ultrasound-measured thickness might not be the thickest as it depends on where the probe laid, it is of great value for surgical planning. Moreover, MRI images could be stored permanently and easily accessed by obstetricians and referenced for further training and research, while in contrast, complete ultrasound images were not readily available to the obstetric surgeons for operative planning, and surgeons had to separately request several screenshots of ultrasound images.

It should also be noted that total hysterectomy results in detrimental effects in many aspects of pelvic floor function [40]. In a review of 11 observational studies, developing urinary incontinence after hysterectomy was about 40% higher than those who have not undergone this procedure [41]. In addition, a profound impact of hysterectomies on sexual function has been reported [42]. Moreover, adverse psychological outcomes including post-traumatic stress disorder (PTSD) can result from emergency postpartum hysterectomies [42–44]. Thus, it stands to reason that a uterus-preserving approach not only suited the cultural needs of the local population, but also avoided adverse impacts on the quality of life frequently associated with hysterectomy.

We understand that our study should have been conducted as a randomized controlled trial study design. However, PAS is a rare life-threatening pregnancy disorder and the Institutional Review Board would not approve randomization when the studied approach is obviously more effective than other approaches. Besides, this study was limited by its retrospective nature, and may not be generalizable to other regions with different cultural norms, and therefore a different cost-benefit ratio with respect to uterus preservation. As no total hysterectomy were performed at this center, we are unable to directly compare patient outcomes and satisfaction between the studied approach and total hysterectomy.

## Conclusions

The current conservative, uterus-preserving management for PAS is safe and effective in the treatment of PAS. Compared with other techniques published in the literature, uterine artery ligation combined with B-Lynch suture was more effective in controlling hemorrhage with a low complication rate. Further research is needed for evaluating long-term outcomes, especially in psychological outcomes.

### Electronic supplementary material

Below is the link to the electronic supplementary material.

Supplementary Material 1

Supplementary Material 2

## Data Availability

The datasets used and/or analyzed during the current study are available from the corresponding author on reasonable request.

## List of abbreviations

CD: cesarean delivery
CST: clover suture technique
MRI: magnetic resonance imaging
PAS: placenta accrete spectrum
UAL: uterine artery ligation
