# Supplementary material for: Uterus-preserving surgical management of placenta accreta spectrum disorder: a large retrospective study
Source: BMC Pregnancy Childbirth. 2023 Aug 26;23:615. doi: 10.1186/s12884-023-05923-9 (PMC10464453; doi:10.1186/s12884-023-05923-9)

**Photos** **of the surgical management approach of UAL+CST**


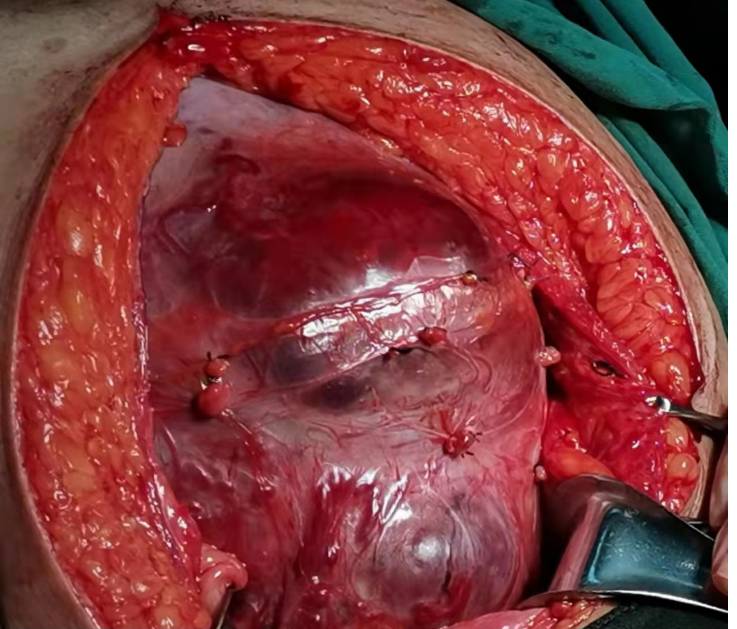
1 taken after opening the abdominal cavity


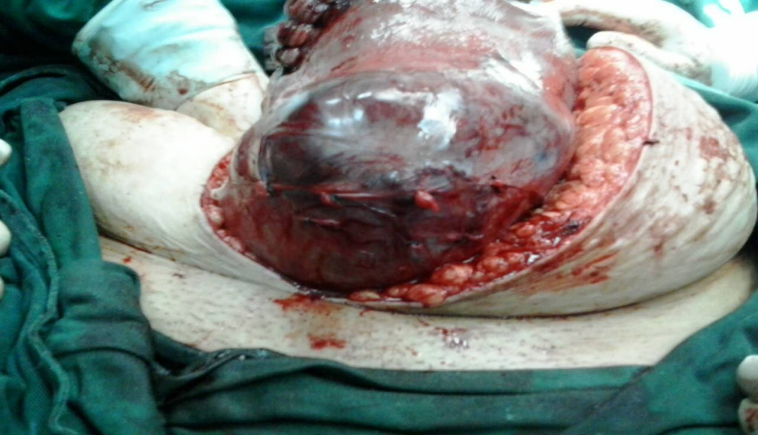

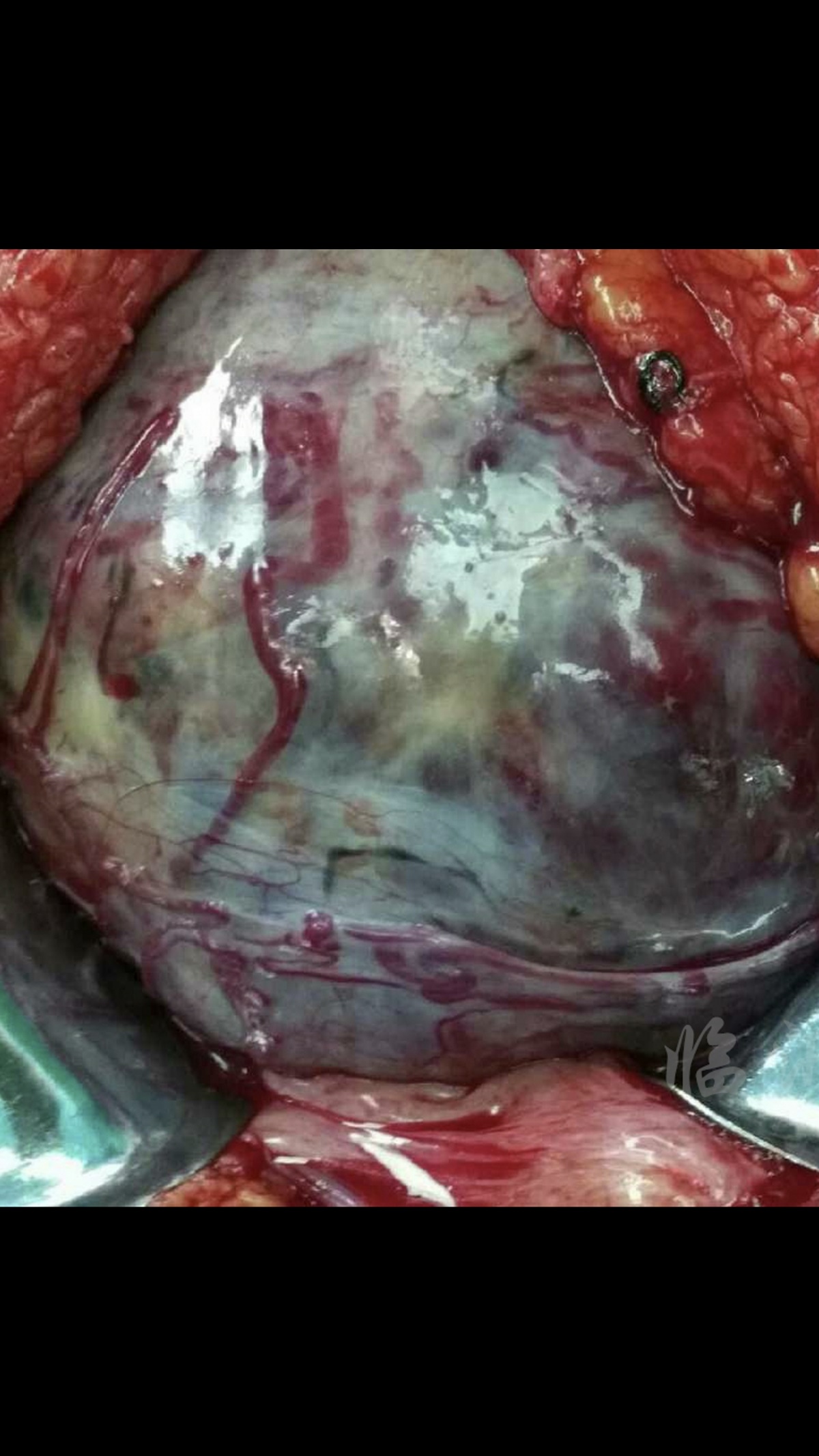

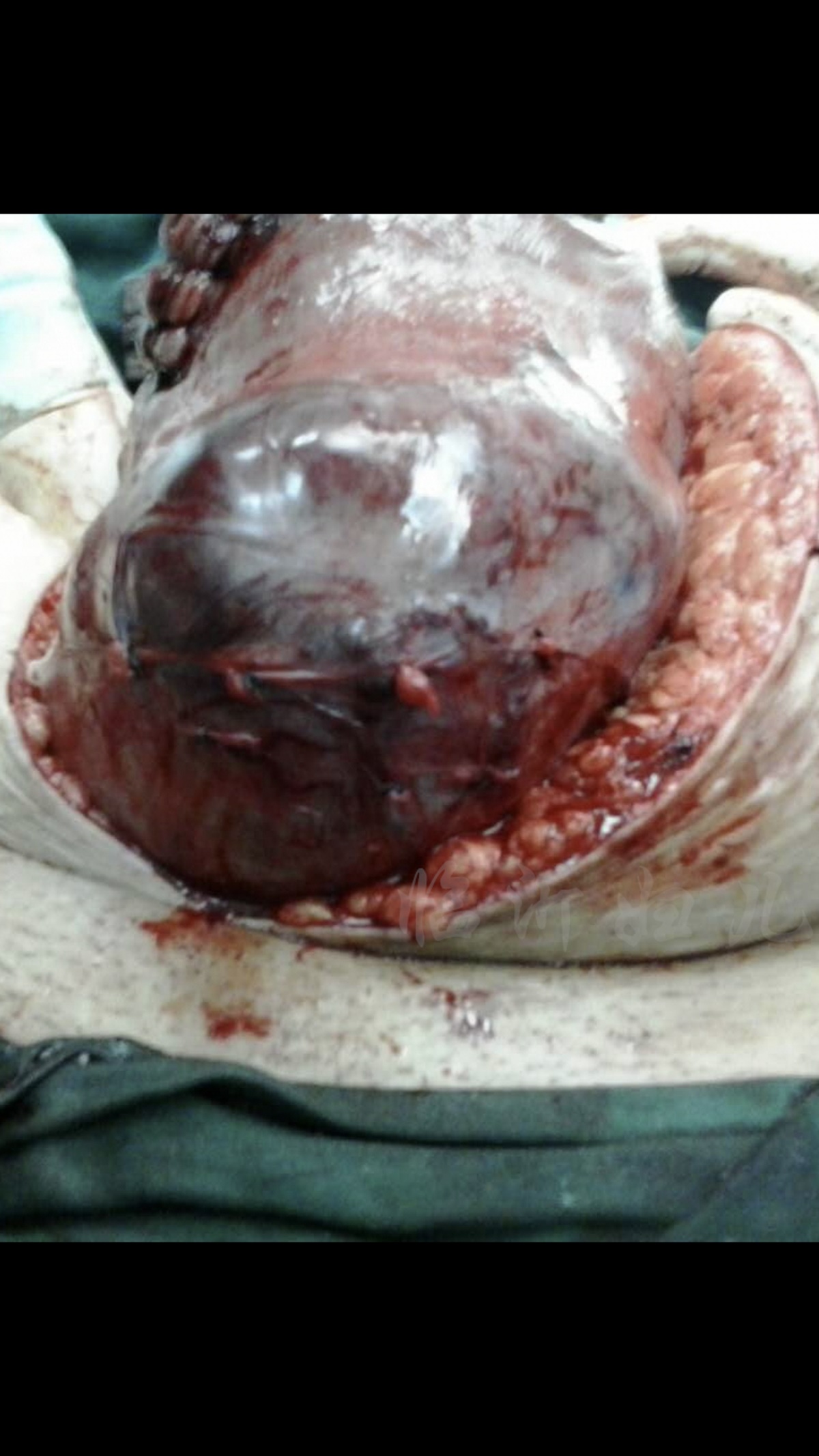

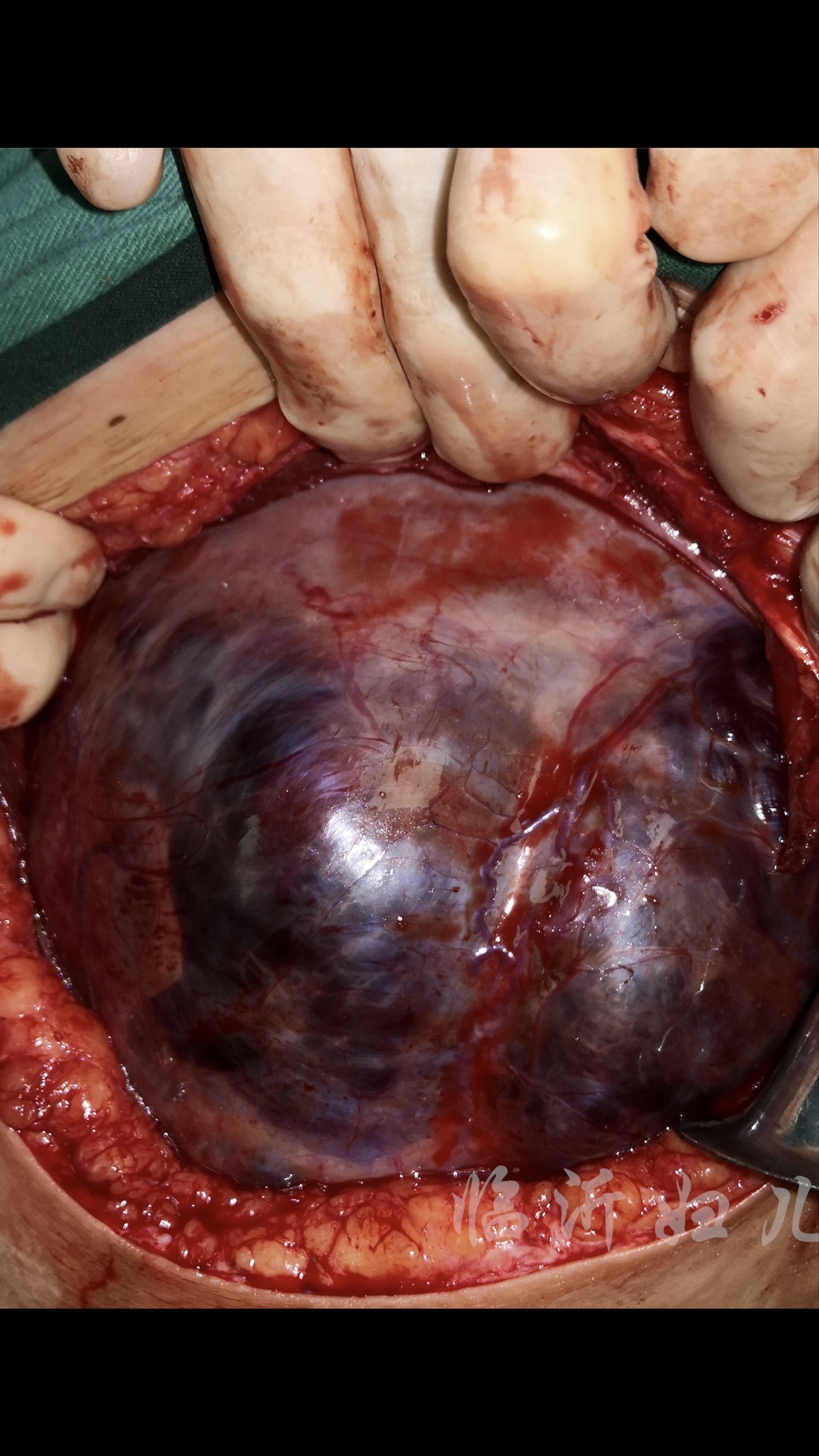

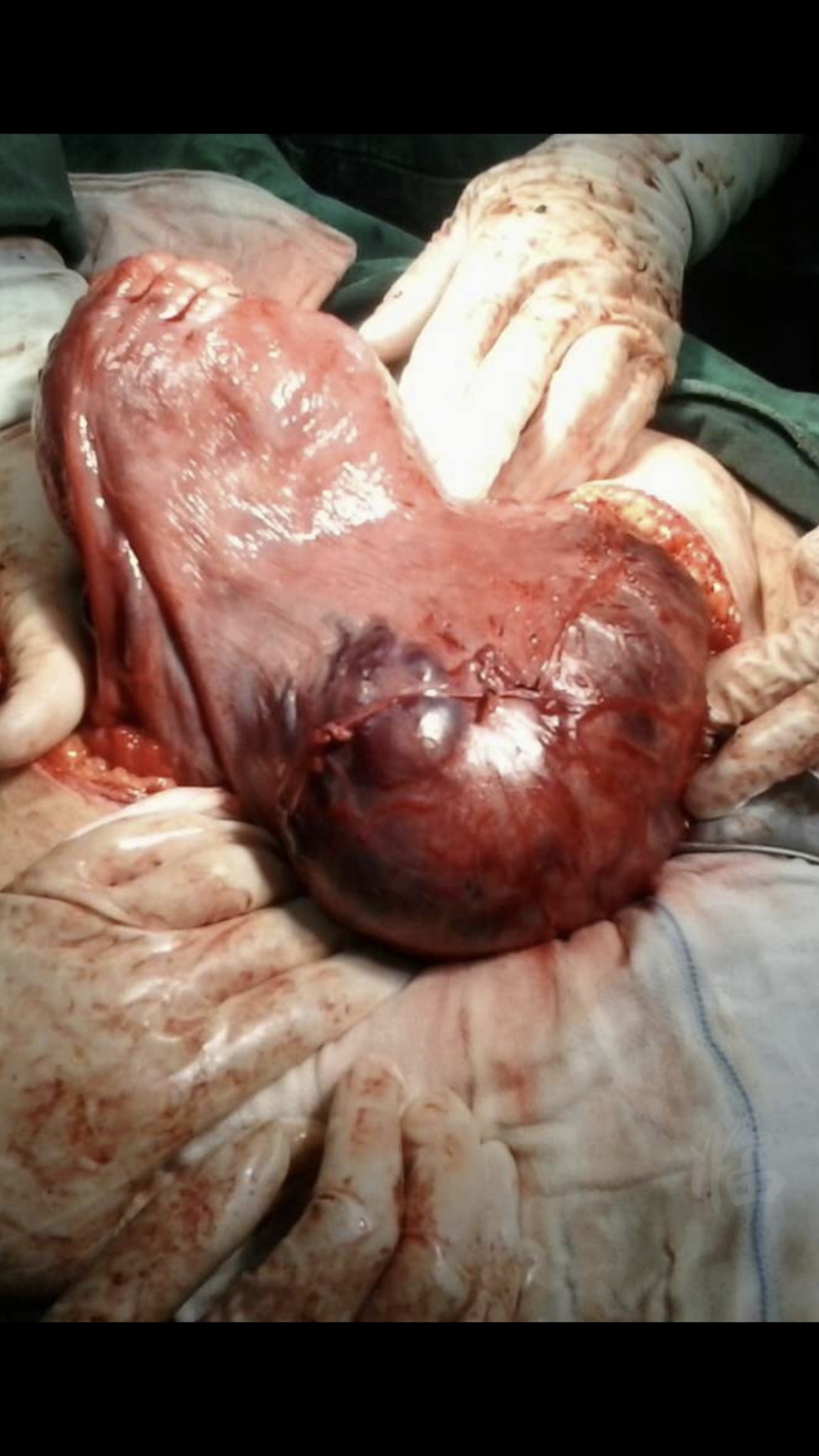

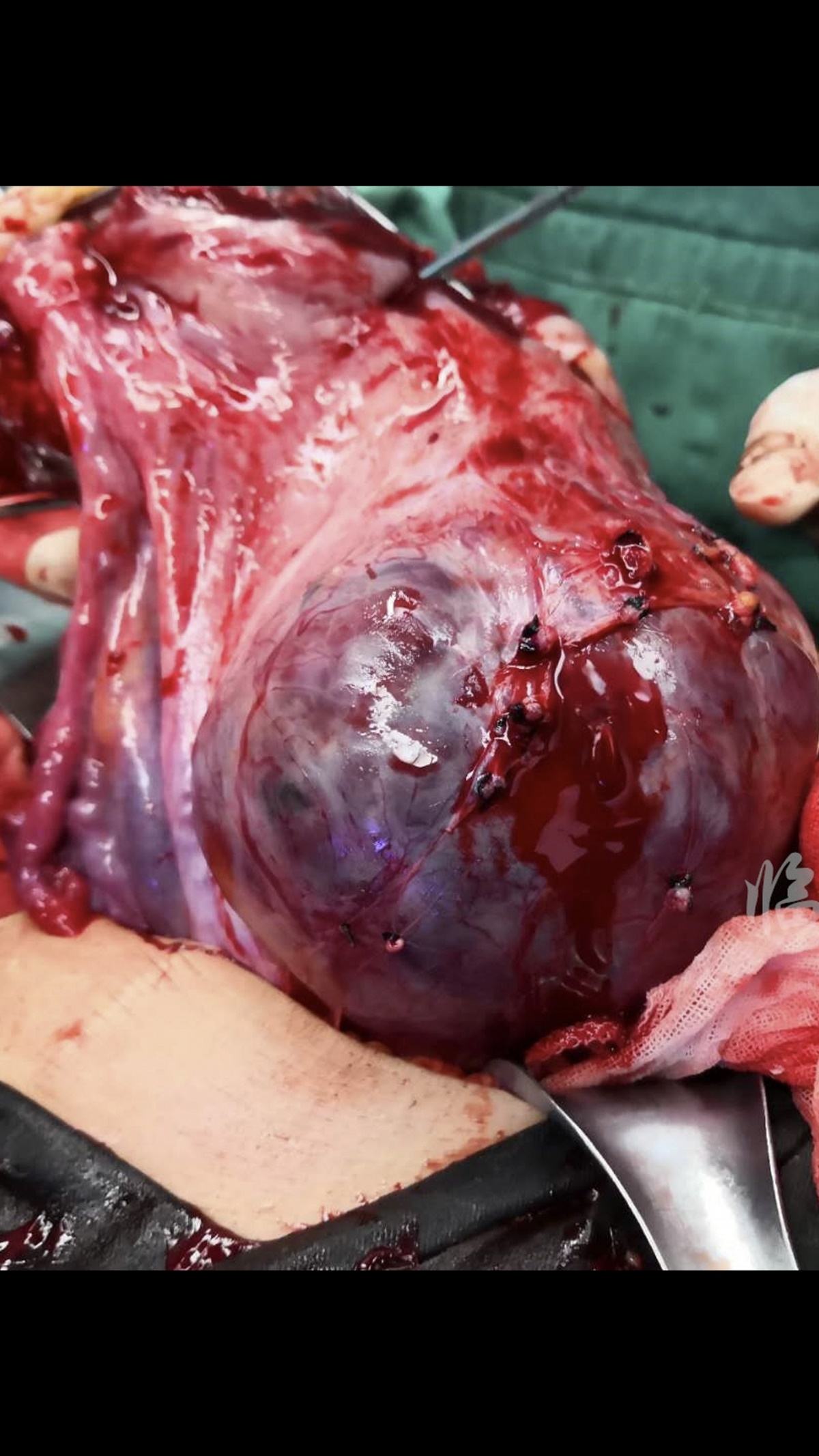

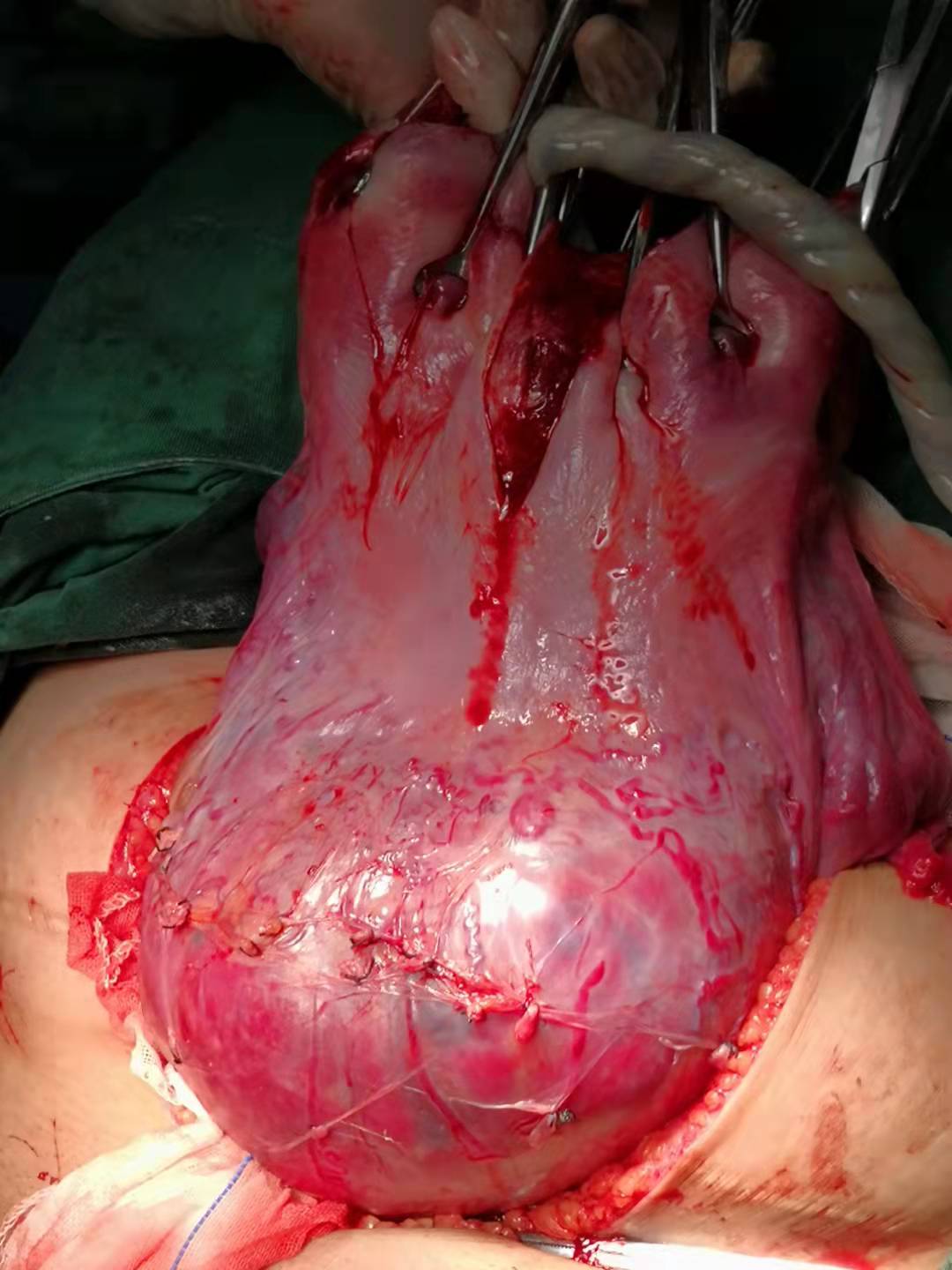

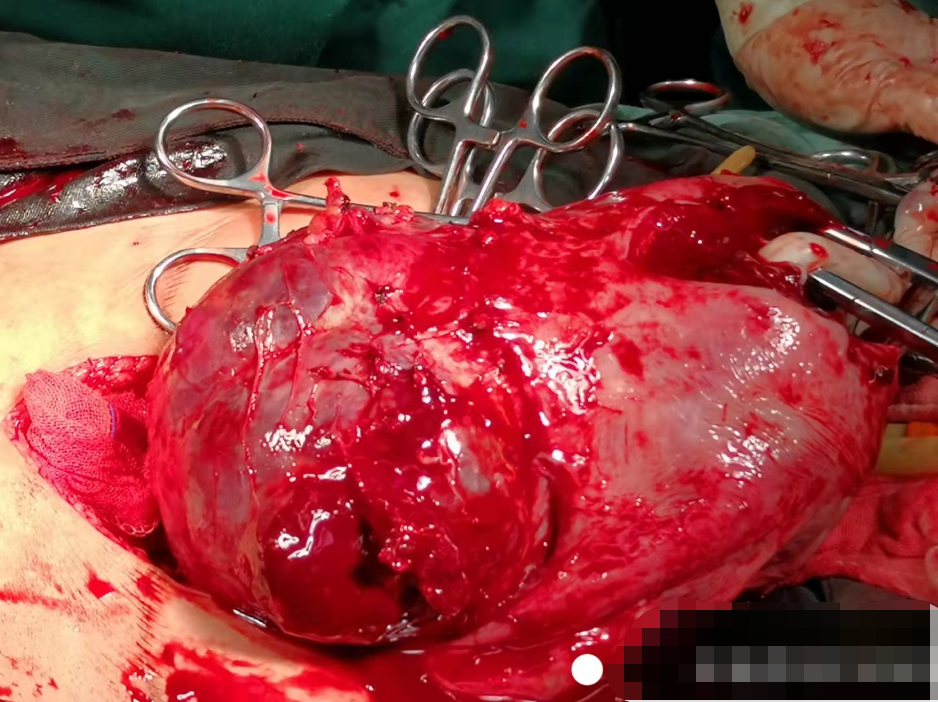

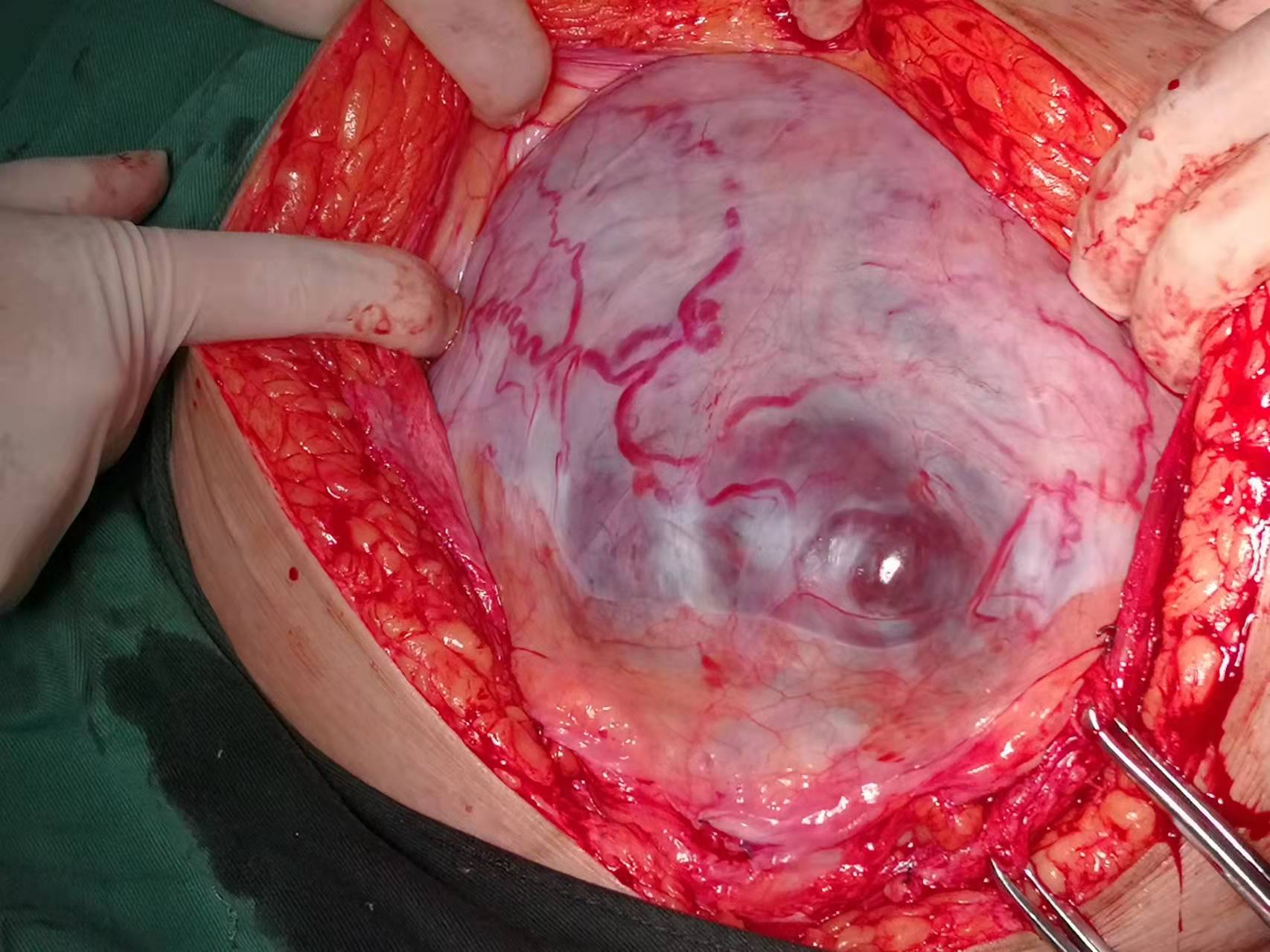

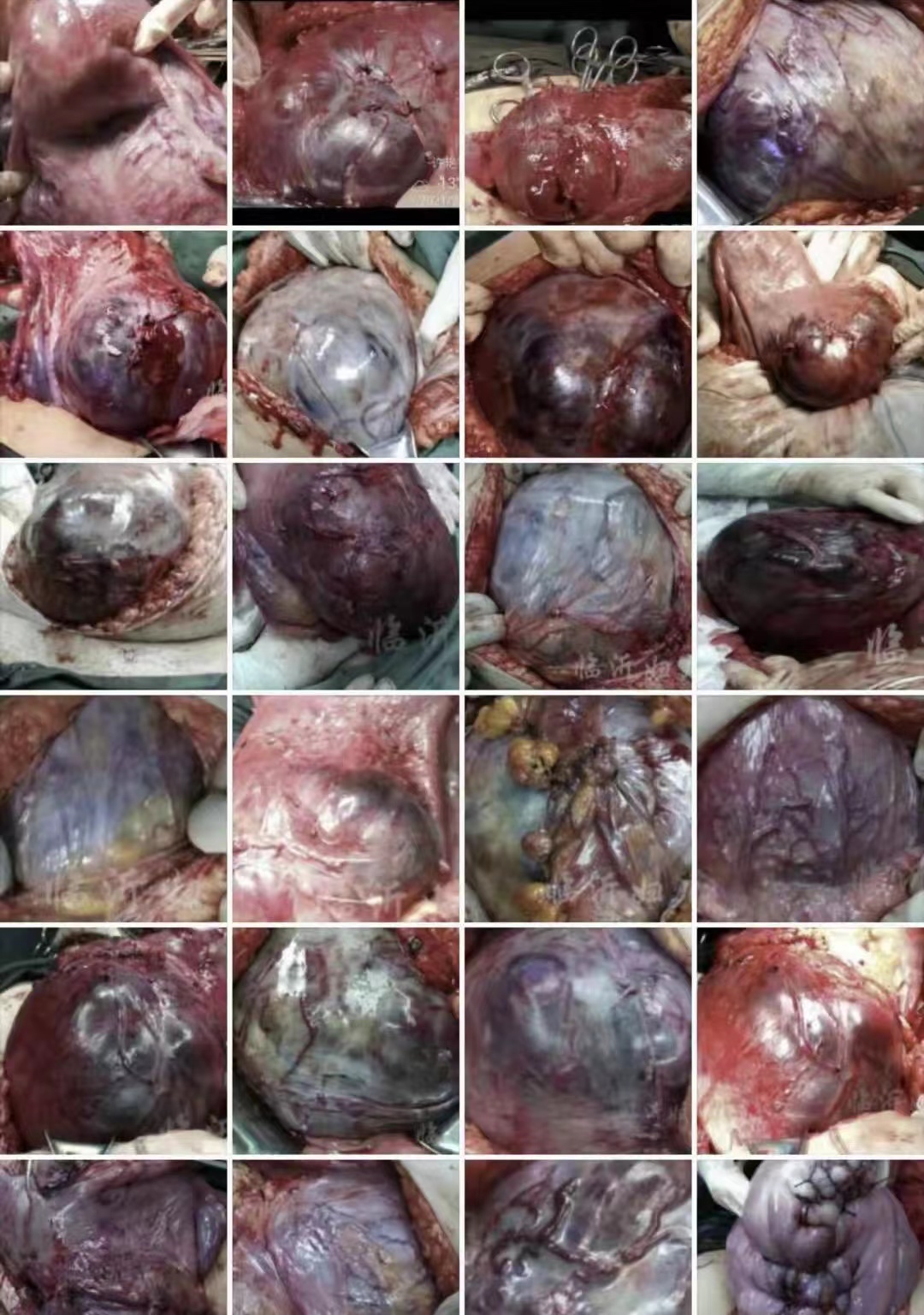


2 taken during the entire operation

2.1 Magnetic resonance imaging (MRI) as an antenatal diagnostic tool


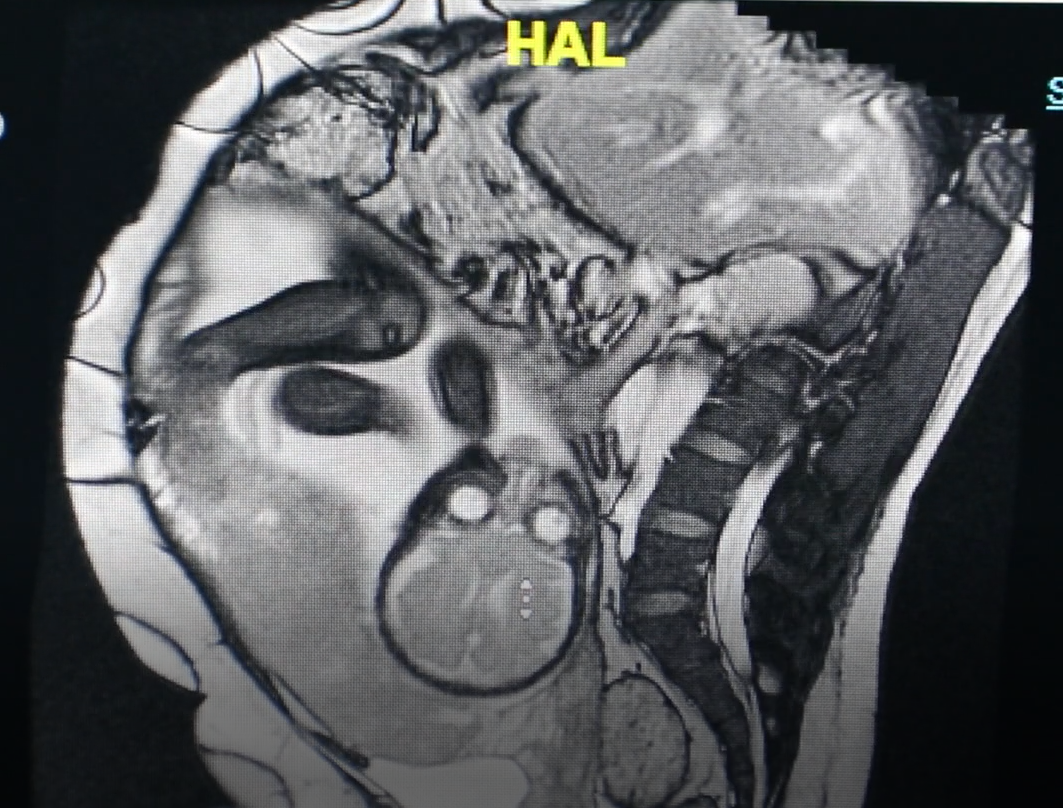


2.2 An infra-umbilical midline incision was made.


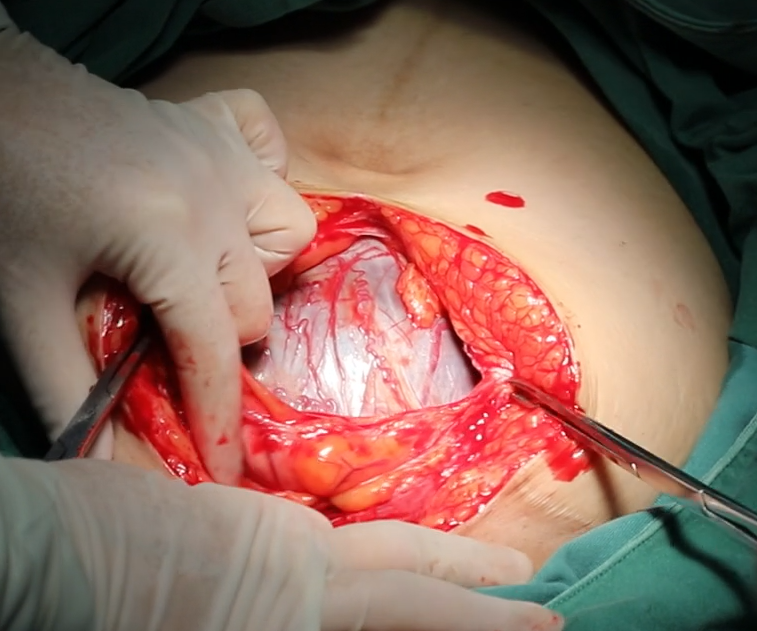


2.3 The bladder was pushed down to ligate invading blood vessels


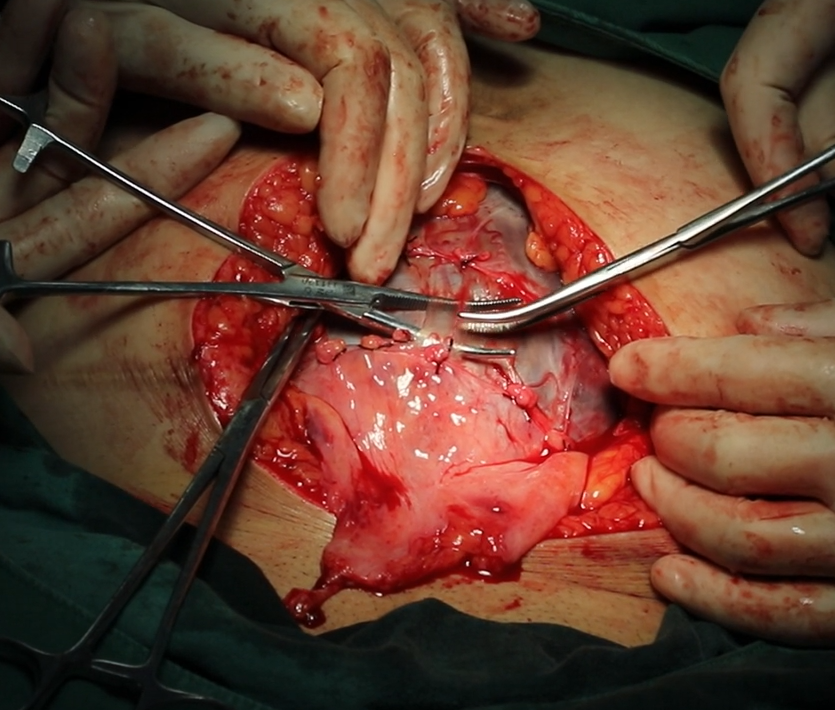


2.4 A vertical or horizontal incision was made on the upper uterus to delivere the fetus


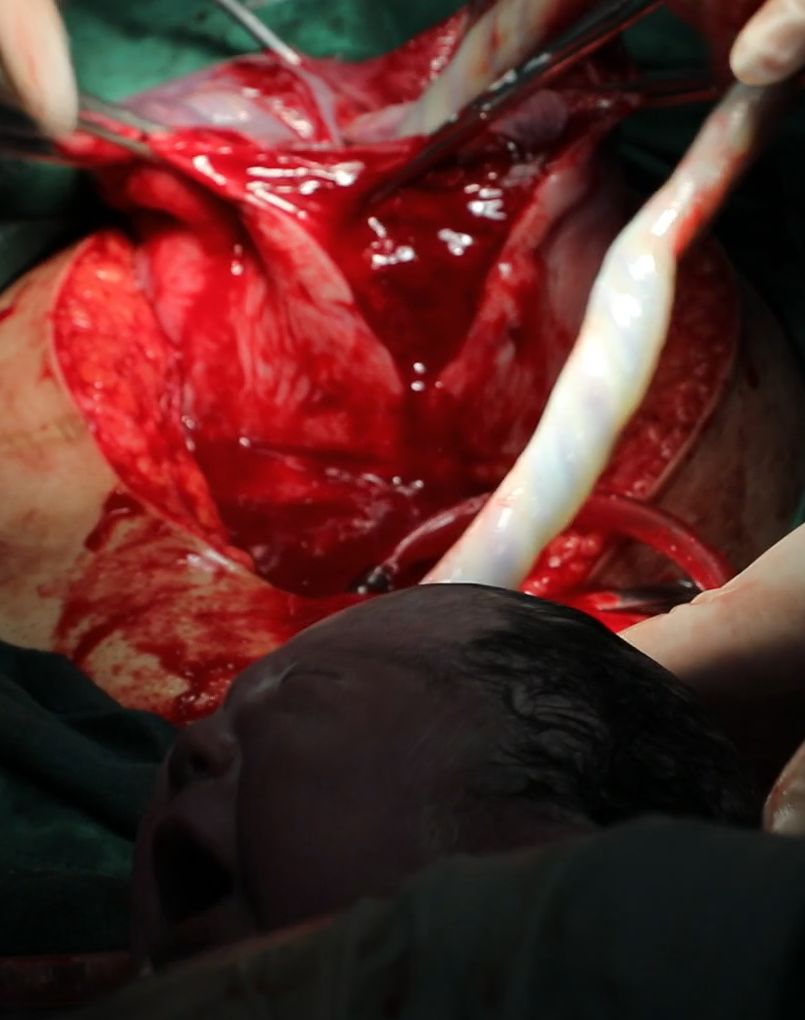


2.5 After delivering the fetus.


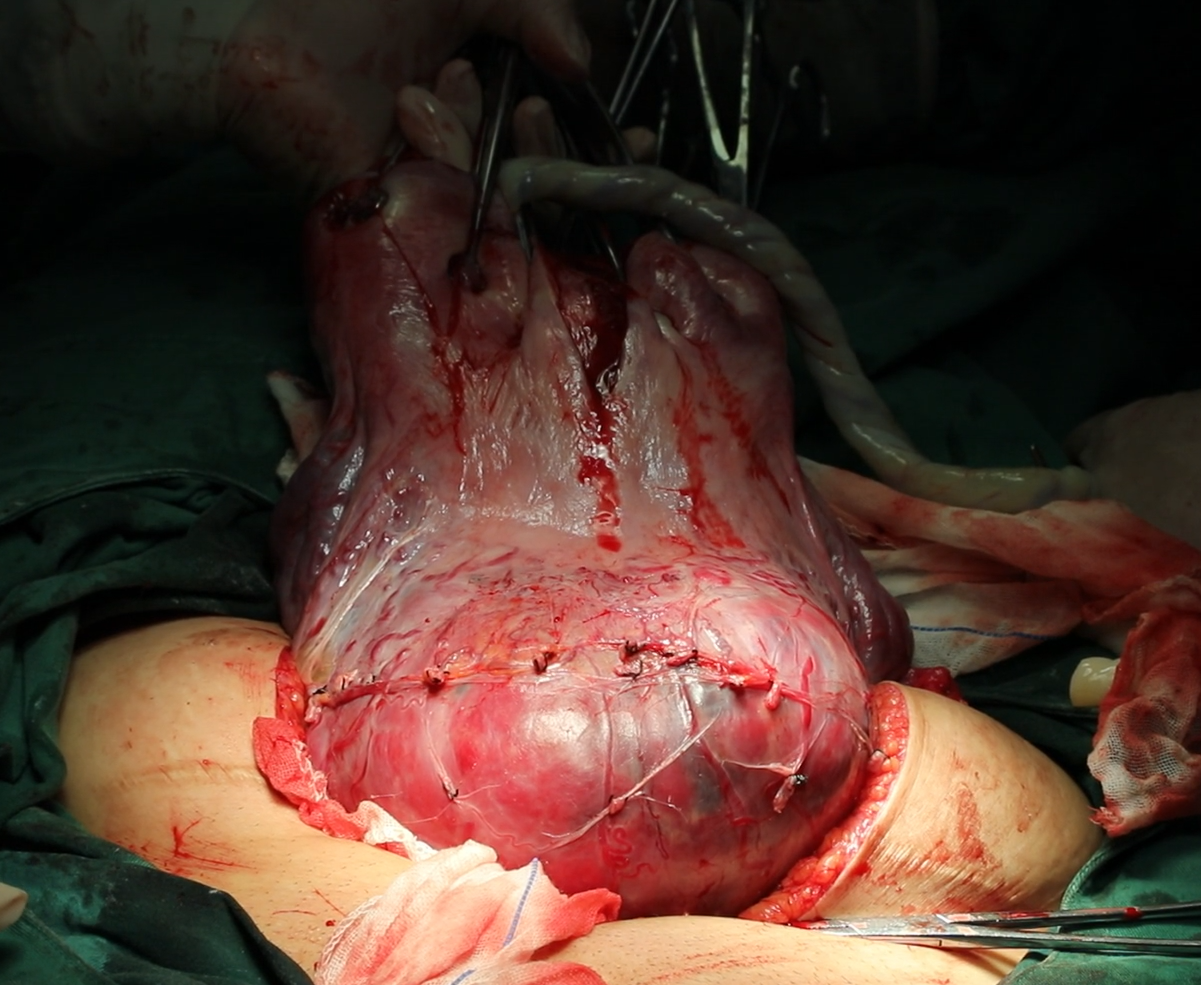


2.6 The ascending branch of uterine artery was ligated(right)）


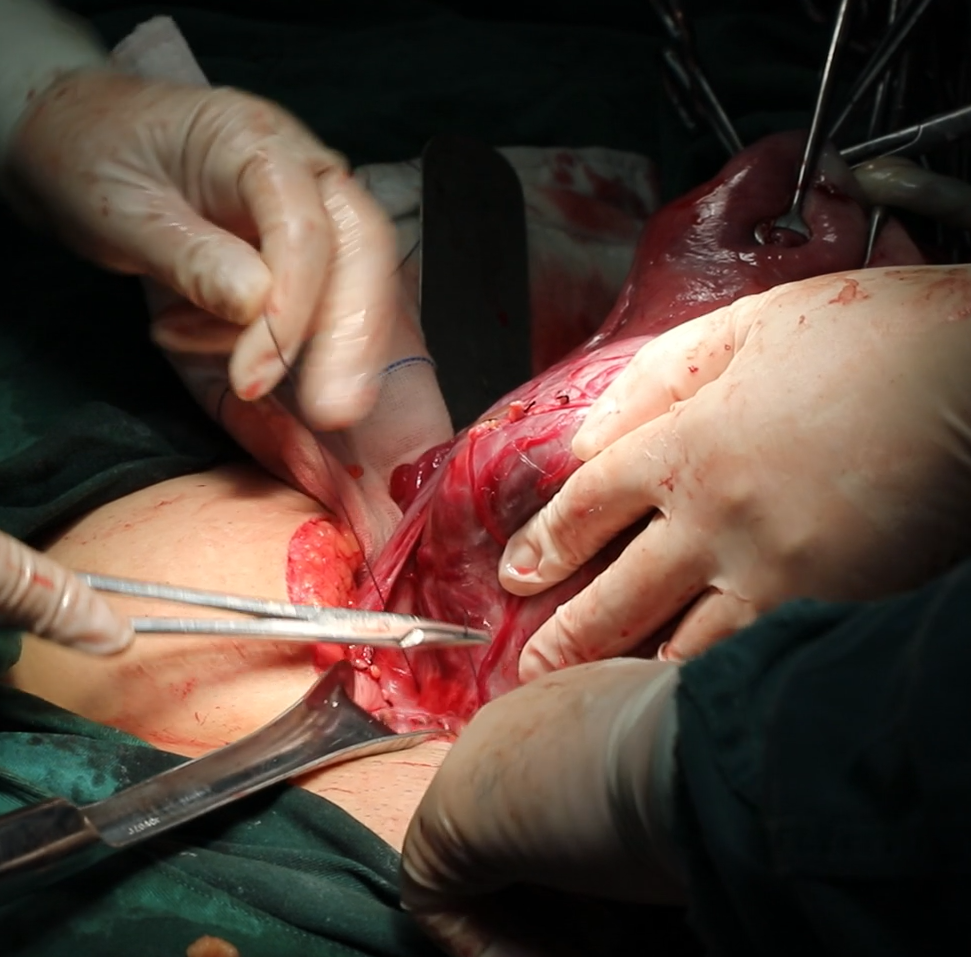


2.7 the ascending branch of uterine artery was ligated(left)


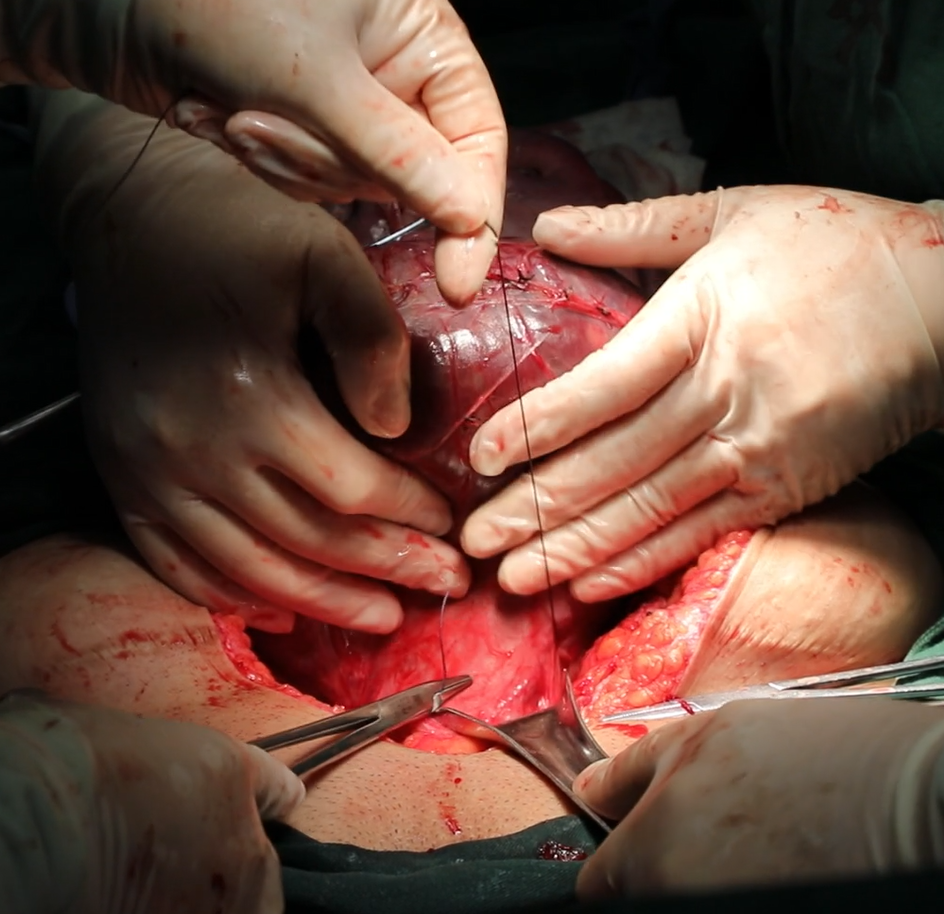


2.8 The first needle：cervix clover


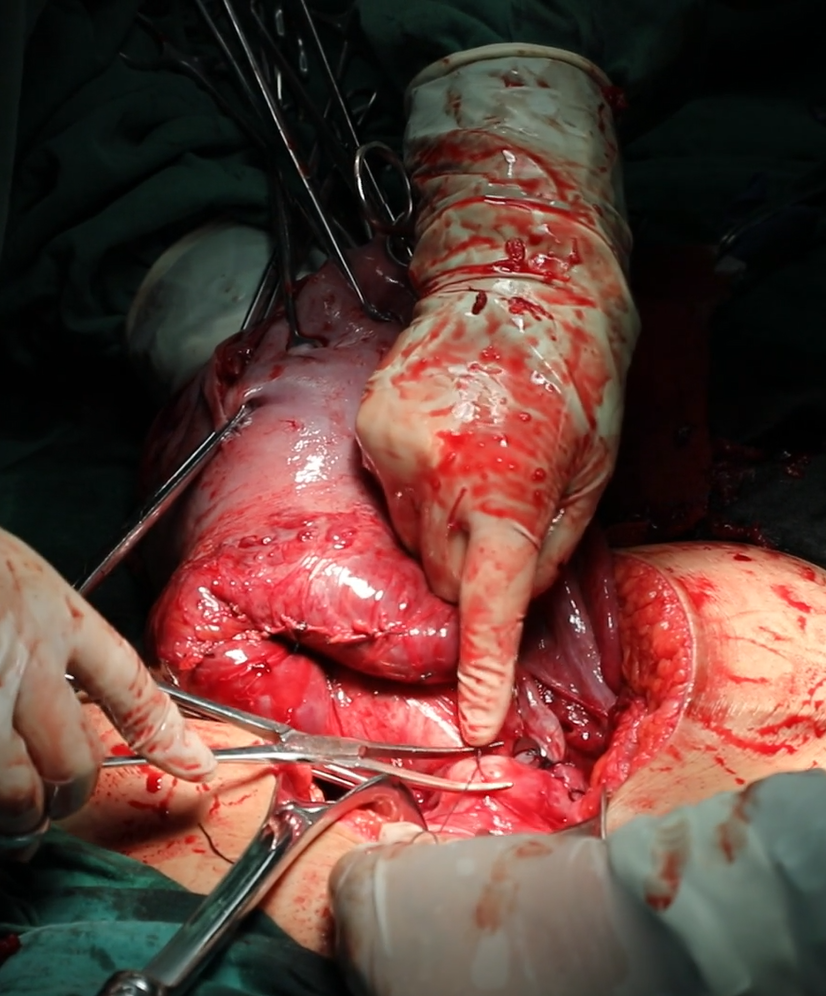


2.9 The second needle：cervix clover


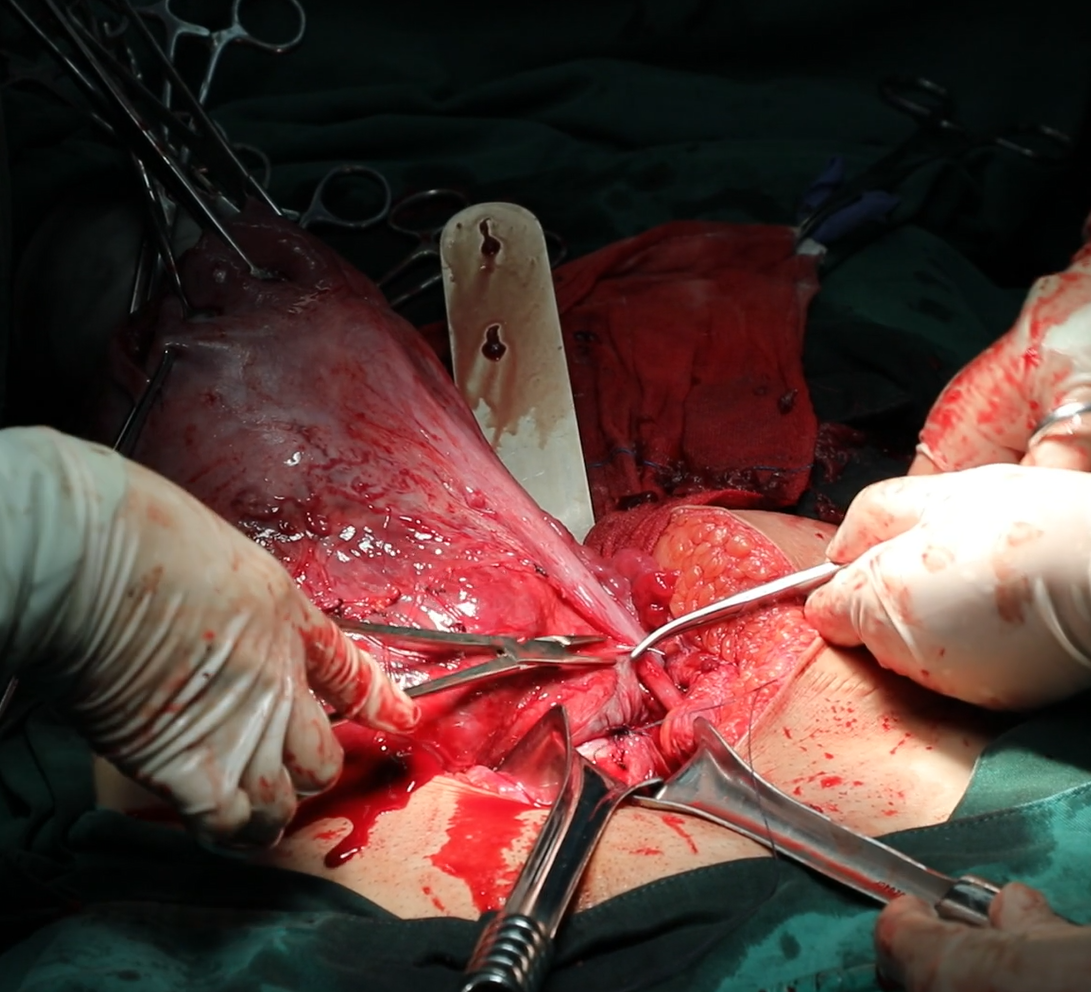


2.10 The third needle：cervix clover


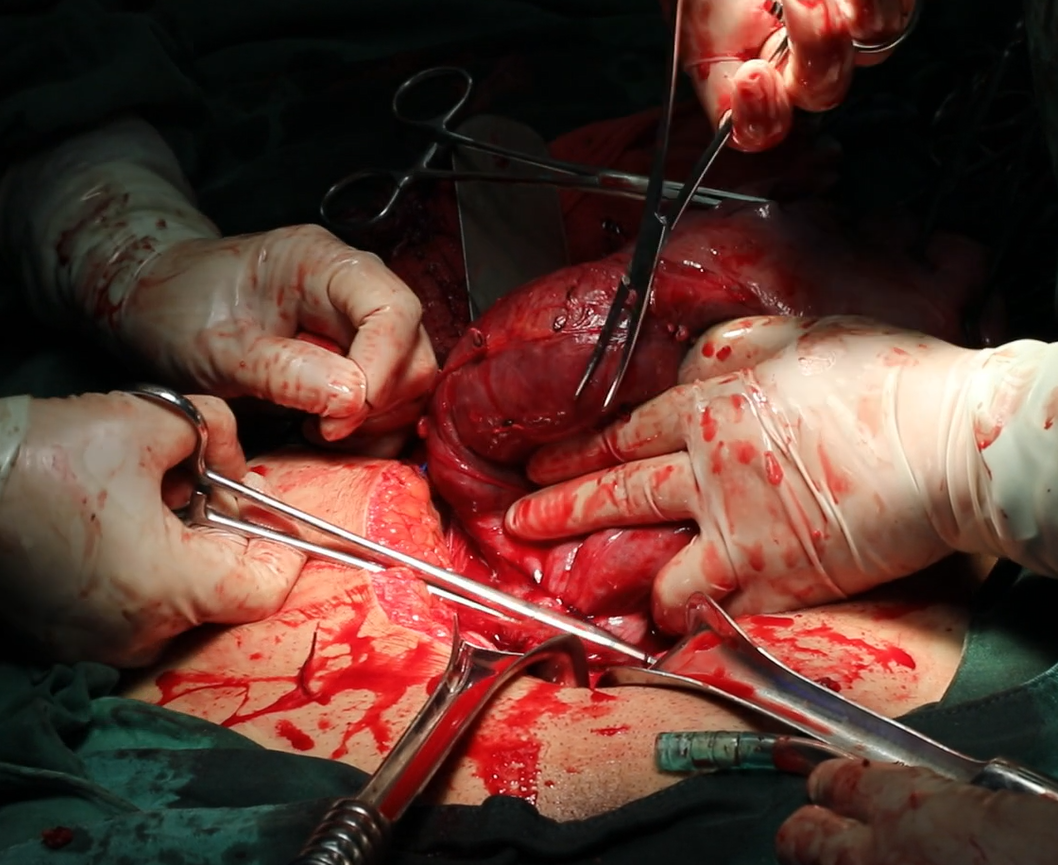


2.11 The fourth needle：lower segment clover


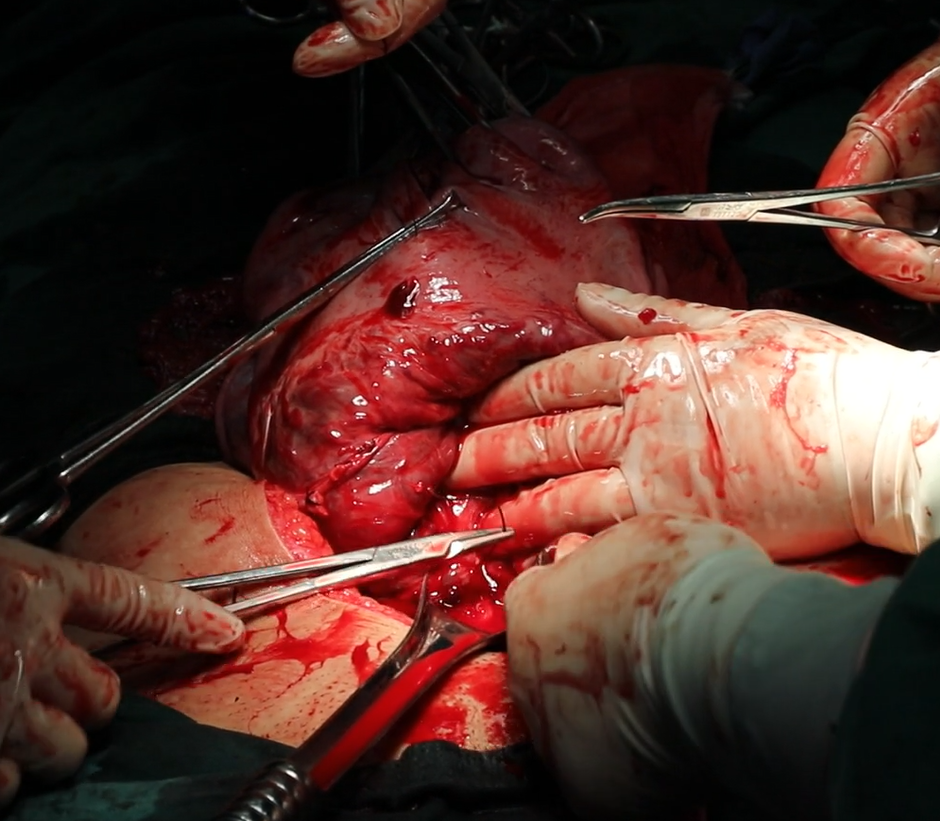


2.12 The fifth needle：lower segment clover


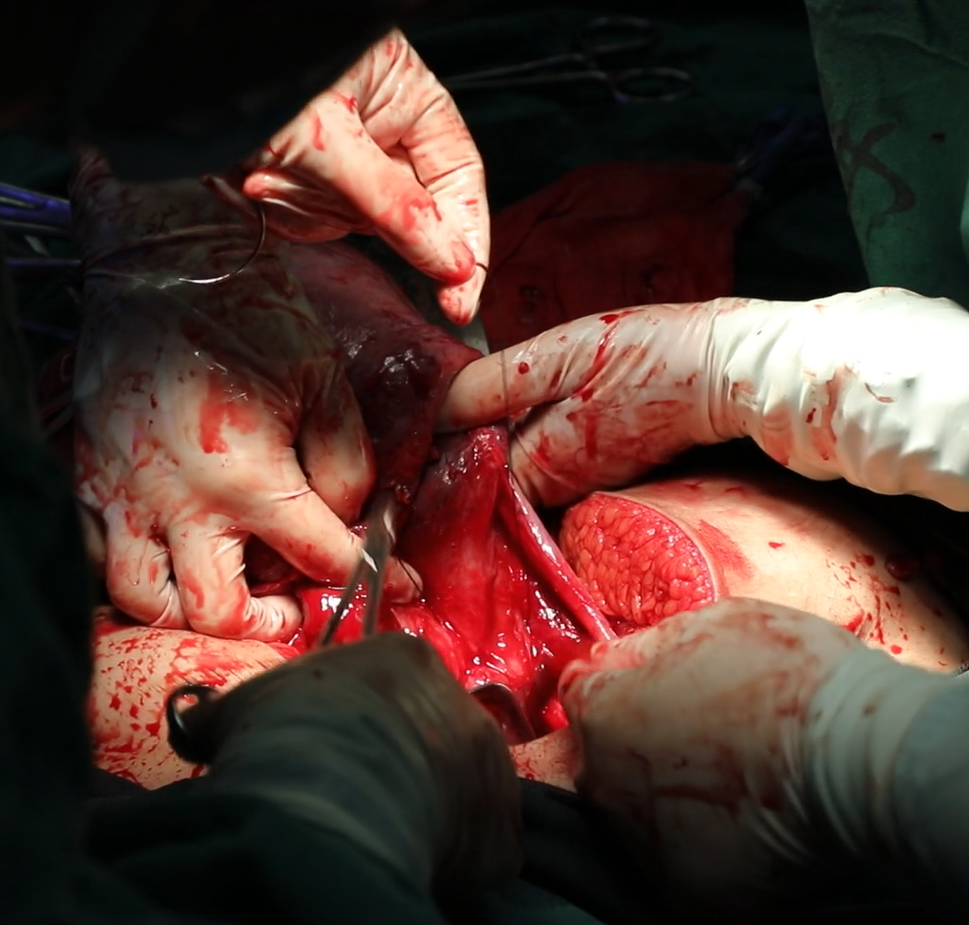


2.13 The sixth needle：lower segment clover


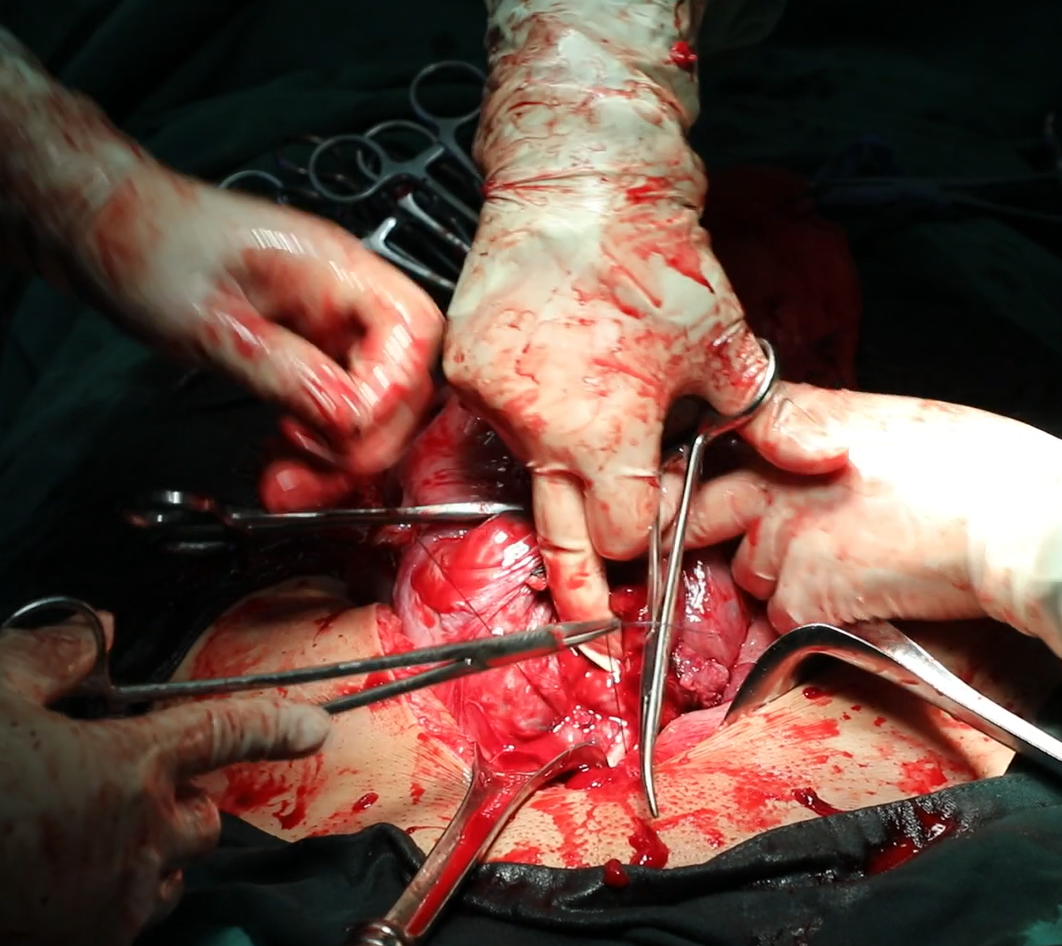


2.14 The end


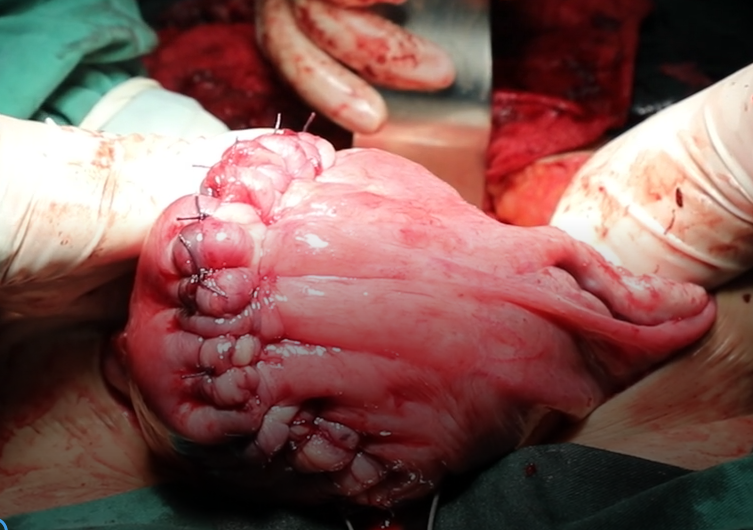


2.15 The end


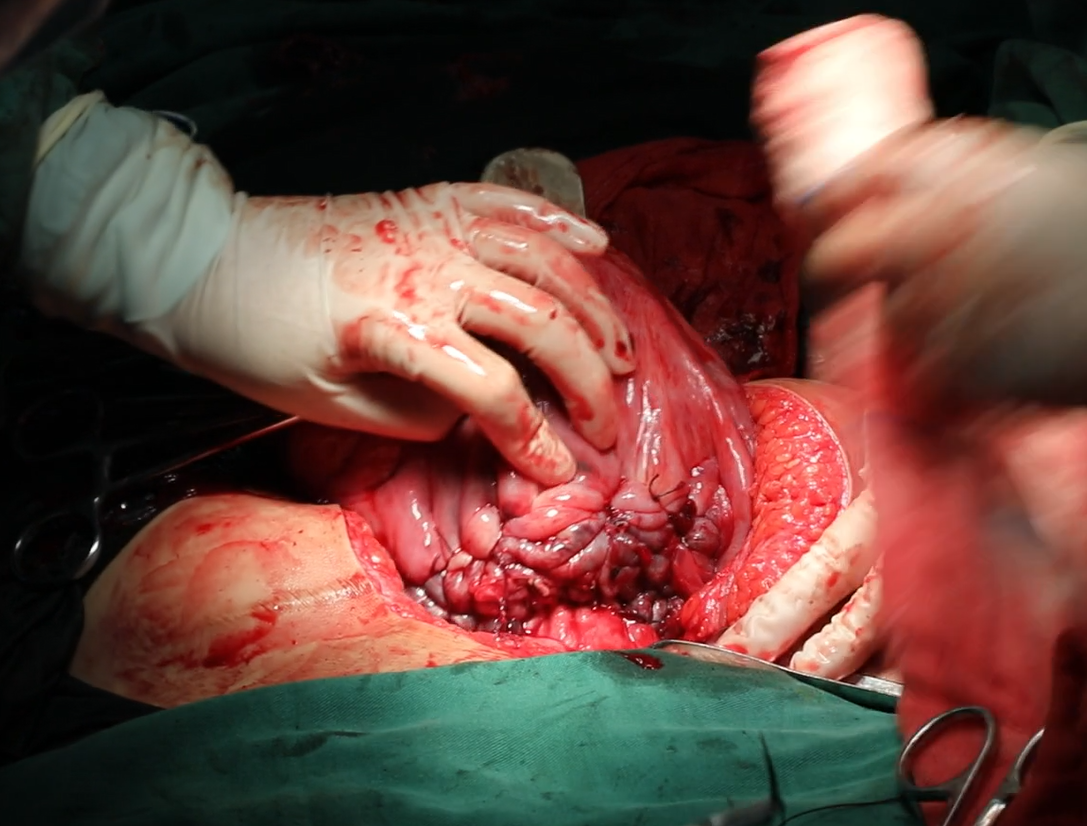

Supplement: Supplementary file 1 — Supplementary Material 1 [file 12884_2023_5923_MOESM1_ESM.docx]
