# Supplementary material for: Uterus-preserving surgical management of placenta accreta spectrum disorder: a large retrospective study
Source: BMC Pregnancy Childbirth. 2023 Aug 26;23:615. doi: 10.1186/s12884-023-05923-9 (PMC10464453; doi:10.1186/s12884-023-05923-9)
Supplement: Supplementary file 2 — Supplementary Material 2 [file 12884_2023_5923_MOESM2_ESM.docx]

**
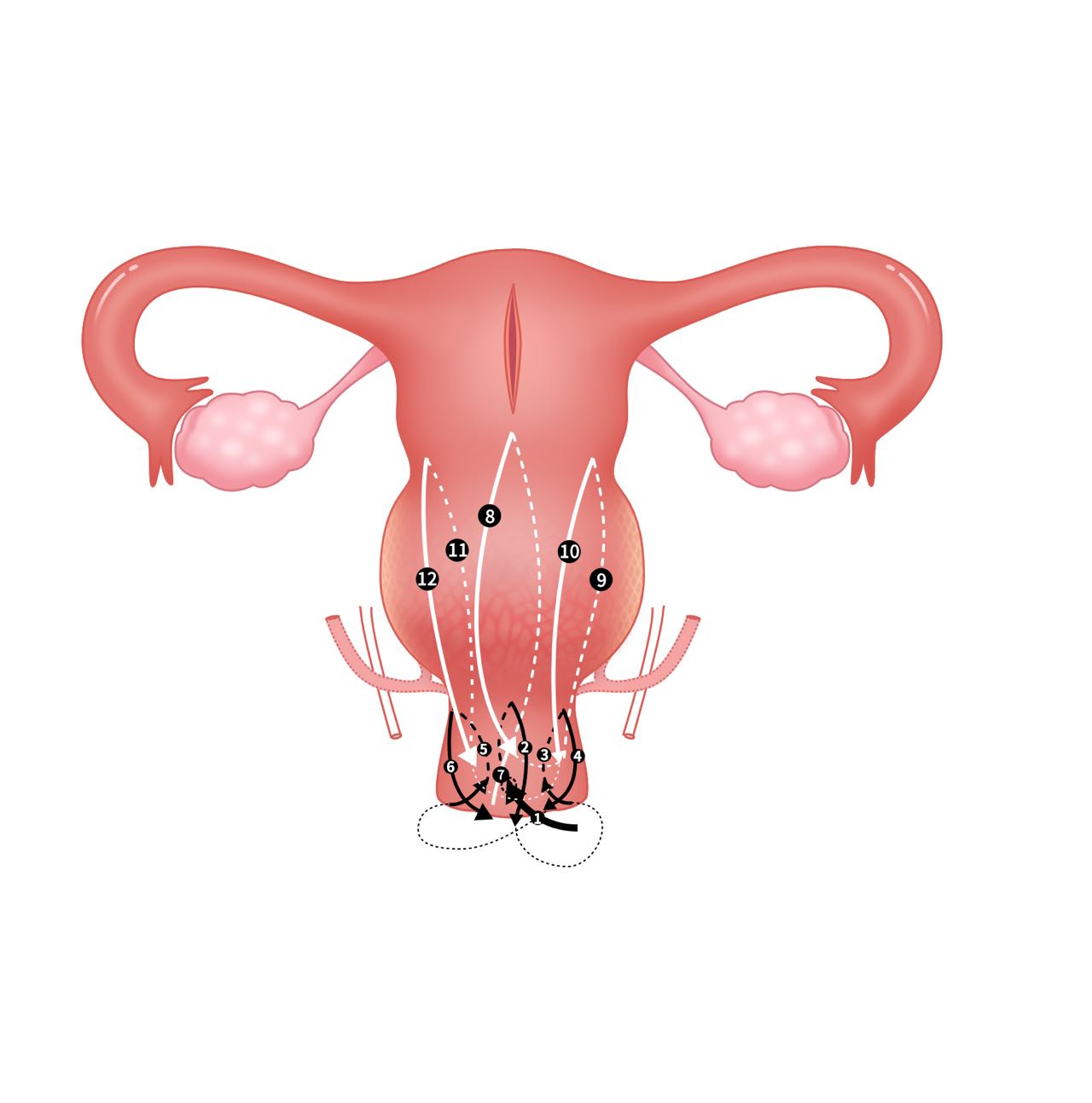
**

**Clover suture technique (CST) – Figure 1 b**

CST was proposed by the team specifically for PAS surgery. It was usually performed on the lower segment of the uterus, and also on the cervix when the placenta invasion reached the cervix. Figure 1 b shows the condition when CST was performed both on the cervix and the lower segment of the uterus.

When placental invasion anomaly reaches the cervix, CST would be performed in cervix, which includes 6 steps, as shown in figure 1 b from step 1 to step 6.

The needle is inserted from the midpoint of the anterior wall of the cervix at the level of the external cervical orifice, and passes through the serosa toward the cervix cavity (first entrance; figure 1 b-➀). The needle moves upward in the cervical cavity to the level of the internal cervical orifice, and exits from the anterior wall of the cervix and knots with the suture tail on the surface of the cervix (first exit; figure 1 b-➁)

The second needle is inserted from near the right side of the entry point of the first needle and passes through the serosa (second entrance; figure 1 b-➂). The needle moves upward to the right at an angle of 45 degrees, then exits after reaching the right margin of the cervix, and knots with the suture tail of the first needle on the surface of the cervix (second exit; figure 1 b-➃).

The third needle is inserted from near the left side of the entry point of the first needle and passes through the serosa (third entrance; figure 1 b-➄). The needle moves upward to the left at an angle of 45 degrees, then exits after reaching the left margin of the cervix, and knots with the suture tail of the first needle on the surface of the cervix (third exit; figure 1 b-➅).

After CST is finished on the cervix, we perform it again in the lower segment of the uterus, which also includes 6 steps, as shown in Supplemental figure from step 7 to step 12.

The fourth needle is inserted from below the first exit of “cervix clover” to overlap the “cervix clover” with the “lower uterine segment clover” for strengthen and support, and passes through the entire uterine wall (serosa, myometrium, and endometrium) toward the uterine cavity (fourth entrance; figure 1 b-➆). The needle moves upward in the uterine cavity toward the normal myometrium level of the uterine body, then exits the needle 1centimeter above the normal myometrium level and knots on the surface of the uterus (fourth exit; figure 1 b-8➇).

The fifth needle is inserted from below the second exit of the “cervix clover” to overlap the “cervix clover” with the “lower uterine segment clover” for strengthen and support, and passes through the entire uterine wall (serosa, myometrium, and endometrium). The needle moves upward to the right uterine cavity margin at an angle of 45 degrees and across the weak area of the anterior wall of the uterus where the placenta was once attached (fifth entrance; figure 1 b-➈). Then the needle exits and knots with the suture tail of the fourth needle on the surface of the uterus (fifth exit; figure 1 b-➉).

The sixth needle is inserted from below the third exit of the “cervix clover” to overlap the “cervix clover” with the “lower uterine segment clover” for strengthen and support, and passes through the entire uterine wall (serosa, myometrium, and endometrium). The needle moves upward to the left uterine cavity margin at an angle of 45 degrees and across the weak area of the anterior wall of the uterus where the placenta was once attached (sixth entrance; figure 1 b-⑪). Then the needle exits and knots with the suture tail of the fourth needle on the surface of the uterus (sixth exit; figure 1 b-⑫).

After both CSTs were finished, several stitches can be added to reinforce as appropriate.
